# Regulation of Active DNA Demethylation by a Methyl-CpG-Binding Domain Protein in *Arabidopsis thaliana*

**DOI:** 10.1371/journal.pgen.1005210

**Published:** 2015-05-01

**Authors:** Qi Li, Xiaokang Wang, Han Sun, Jun Zeng, Zhendong Cao, Yan Li, Weiqiang Qian

**Affiliations:** State Key Laboratory of Protein and Plant Gene Research, The Peking-Tsinghua Center for Life Sciences, School of Advanced Agricultural Sciences and School of Life Sciences, Peking University, Beijing, China; Indiana University, Howard Hughes Medical Institute, UNITED STATES

## Abstract

Active DNA demethylation plays crucial roles in the regulation of gene expression in both plants and animals. In *Arabidopsis thaliana*, active DNA demethylation is initiated by the ROS1 subfamily of 5-methylcytosine-specific DNA glycosylases via a base excision repair mechanism. Recently, IDM1 and IDM2 were shown to be required for the recruitment of ROS1 to some of its target loci. However, the mechanism(s) by which IDM1 is targeted to specific genomic loci remains to be determined. Affinity purification of IDM1- and IDM2- associating proteins demonstrated that IDM1 and IDM2 copurify together with two novel components, methyl-CpG-binding domain protein 7 (MBD7) and IDM2-like protein 1 (IDL1). IDL1 encodes an α-crystallin domain protein that shows high sequence similarity with IDM2. MBD7 interacts with IDM2 and IDL1 *in vitro* and *in vivo* and they form a protein complex associating with IDM1 *in vivo*. MBD7 directly binds to the target loci and is required for the H3K18 and H3K23 acetylation *in planta*. *MBD7* dysfunction causes DNA hypermethylation and silencing of reporter genes and a subset of endogenous genes. Our results suggest that a histone acetyltransferase complex functions in active DNA demethylation and in suppression of gene silencing at some loci in *Arabidopsis*.

## Author Summary

DNA cytosine methylation (5-methylcytosine, 5-meC) plays important roles in genome organization, genomic imprinting, transposon silencing and gene expression. DNA methylation patterns are dynamically controlled by methylation and demethylation reactions during development and reproduction in eukaryotes. In *Arabidopsis thaliana*, ROS1, a 5-methylcytosine DNA glycosylase, is responsible for active DNA demethylation via a base excision repair pathway. Our previous work showed that a histone acetyltransferase, IDM1, is an important regulator of active DNA demethylation. Here we identified two components, IDL1 and MBD7, as additional novel regulators of active DNA demethylation. Our data suggest that a histone acetyltransferase complex, composed of MBD7, IDL1, IDM2 and IDM1, is required for the recruitment of ROS1 to specific loci for active DNA demethylation. Our findings significantly expand our understanding of the regulatory mechanisms underlying active DNA demethylation in plants.

## Introduction

DNA methylation is an important epigenetic mark conserved in many eukaryotes. Many studies have demonstrated that DNA methylation plays central roles in genome organization, genomic imprinting, transposon silencing, and gene expression [1,2,3]. DNA methylation levels are coordinately determined by methylation and demethylation reactions during development and reproduction in both plants and animals. In the model plant *Arabidopsis thaliana*, levels of symmetric CG and CHG methylation are maintained by DNA METHYLTRANSFERASE 1 (MET1) and CHROMOMETHYLASE 3 (CMT3), respectively, during DNA replication [1]. In contrast, asymmetric CHH methylation needs to be established *de novo* during each cell cycle [1]. DNA methylation is antagonized by an active DNA demethylation pathway catalyzed by a subfamily of bi-functional DNA glycosylase/lyases represented by REPRESSOROF SILENCING 1 (ROS1) and DEMETER (DME) [4,5,6,7,8].

Although the RNA-directed DNA methylation pathway and its regulation are well-established (Law and Jacobsen, 2010), little is known about the locus-specific targeting of proteins involved in active DNA demethylation in plants. Previous studies suggested that the RNA-binding protein ROS3 is required for the recruitment of ROS1 to some target loci [9]. INCREASED DNA METHYLATION 1(IDM1), a histone acetyltransferase, was recently identified as another important regulator of DNA demethylation that functions upstream of ROS1 [10]. With the assistance of IDM2, an alpha-crystallin domain protein, IDM1 recognizes chromatin regions that are marked by CG methylation and low levels of histone 3 lysine 4 (H3K4) and arginine 2 (H3R2) methylation and subsequently catalyzes H3K18 and H3K23 acetylation, which triggers ROS1-mediated DNA demethylation [10,11]. However, the molecular mechanism(s) by which IDM1 is targeted to these specific genomic loci remains largely unknown.

In plants and animals, a set of proteins containing a methyl-CpG-binding domain (MBD) are capable of specifically recognizing and binding methylated DNA [12,13]. They are believed to function as an interpreter of DNA methylation signals [13]. In mammals, MBD proteins MeCP2, MBD1, MBD2 and MBD4 have methyl-CpG-binding activity and function in targeting of maintenance DNA methylation machinery, H3K9 methylation, transcriptional silencing and X chromosome inactivation [14]. They usually execute their functions by binding to other proteins. For example, MeCP2 was demonstrated to interact with Sin3 and histone deacetylases to transcriptionally silence methylated chromosome, suggesting a direct relationship between histone modification and methylated DNA [15]. In *Zebrafish*, MBD4, a protein that possesses DNA glycosylase activity, was reported be involved in active DNA demethylation [16].

The *Arabidopsis* genome contains 13 genes encoding putative MBD proteins orthologous to their mammalian counterparts [17]. Only AtMBD5, AtMBD6 and AtMBD7 bind methylated CpG nucleotide *in vitro* [17]. AtMBD5, AtMBD6 and AtMBD7 localize in the nuclei, and assemble at highly methylated chromocenters. while, in DNA hypomethylation mutants *ddm1* and *met1* they dissociate from chromocenters, indicating their methyl-CpG-binding activity *in vivo* [18]. Different from AtMBD7, AtMBD5 and AtMBD6 mainly localize to chromatin regions covered by ribosomal DNA (rDNA) gene clusters [18,19]. Snf2 family nucleosome remodeler DDM1 is able to bind AtMBD5 and AtMBD6 *in vitro* and facilitate their heterochromatin localization [18]. AtMBD7, belonging to a unique class of plant MBD proteins that have multiple MBD domains, was hypothesized to bind multiple methylated sites that are not close to each other and promote the formation and/or maintainance of a microenvironment in chromatin [13].

To find out how IDM1 is targeted to specific loci, we identified IDM1 and IDM2 interacting proteins by an approach that combines affinity purification and mass spectrometry. We found that IDM1 and IDM2 copurify with each other and with two novel proteins, IDL1 and MBD7. MBD7 directly binds to the target loci and is required for the H3K18 and H3K23 acetylation *in planta*. The *mbd7* mutants exhibit DNA hypermethylation at thousands of genomic loci and show increased transcriptional silencing of reporter genes and some endogenous genes. Our results reveal novel components involved in DNA demethylation and novel functions for MBD proteins in plants.

## Results

### Affinity purification and mass-spectrometric identification of IDM1 and IDM2 interacting proteins

To better understand the mechanism of IDM1 targeting in active DNA demethylation, we set out to purify the protein complex associated with IDM1. Transgenic *Arabidopsis* plants expressing *IDM1-3HA-YFP* driven by native *IDM1* promoter in *idm1-3* mutant background were generated. Previous studies showed that *IDM1* dysfunction caused the kanamycin sensitive growth phenotype and silencing of *NPT II* transgene [20]. The *IDM1-3HA-YFP* was able to complement the kanamycin sensitive growth phenotype (S1A Fig). Real time PCR results showed that the *NPT II* expression was also restored in the transgenic plants (S1B Fig), suggesting that the IDM1-3HA-YFP fusion protein was functional *in vivo*. The IDM1-3HA-YFP expression in the transgenic lines was confirmed by Western blot with the HA antibody (S1C Fig). Co-immunoprecipitation (co-IP) of IDM1-3HA-YFP and its interacting proteins was performed using anti-HA antibodies and flower extracts. Tryptic peptides derived from the purified proteins were sequenced by Velos Pro Orbitrap Elite tandem mass spectrometry (MS/MS) (Table 1 and S1 Table). As expected, the MS analysis identified peptides corresponding to a known IDM1 interacting protein IDM2 [11]. Peptides corresponding to the IDM2-related protein IDM2 like 1 (IDL1) and methyl-CpG-binding domain protein 7 (MBD7) were also identified (Table 1), suggesting these two proteins may be new components of active DNA demethylation pathway. Meanwhile, we carried out affinity purification of 6Myc-IDM2 using transgenic plants overexpressing 6Myc-IDM2 in *idm2-1* mutants [11] and 6Myc-IDL1 using transgenic plants overexpressing 6Myc-IDL1 in wild type plants. Mass spectrometric analysis revealed that IDM2 and IDL1 copurify with each other. We also identified a large number of peptides corresponding to IDM1 and MBD7 (Table 1). Our data suggest that IDM1, IDM2, IDL1, and MBD7 form a tight complex.

**Table 1:** Mass-spectrometric analysis of IDM1, IDM2 and IDL1 co-purifying proteins.

IDM1 Purification								
**Protein Accession**	**Gene Accession**	**Protein**	**Score**	**Mass**	**Spectra**	**Unique Peptides**	**% Seq. Cov**	**% of IDM1**
IPI00540986	AT3G14980	IDM1	4599	133697	236	52	39	100
IPI00545650	AT1G20870	IDL1	925	52470	36	16	39.7	81.5
IPI00524605	AT5G59800	MBD7	749	35127	40	12	40.8	83.7
IPI00531355	AT1G54840	IDM2	531	39712	42	12	37.2	80.8
IDM2 Purification								
**Protein Accession**	**Gene Accession**	**Protein**	**Score**	**Mass**	**Spectra**	**Unique Peptides**	**% Seq. Cov**	**% of IDM2**
IPI00531355	AT1G54840	IDM2	4635	39712	247	27	53.6	100
IPI00540986	AT3G14980	IDM1	3621	133697	162	43	35.4	47.3
IPI00545650	AT1G20870	IDL1	970	52470	39	16	40.4	39.2
IPI00524605	AT5G59800	MBD7	484	35127	22	12	35.3	50.2
IDL1 (IDM2 Like 1) Purification								
**Protein Accession**	**Gene Accession**	**Protein**	**Score**	**Mass**	**Spectra**	**Unique Peptides**	**% Seq. Cov**	**% of IDL1**
IPI00545650	AT1G20870	IDL1	3454	52470	159	36	62.6	100
IPI00540986	AT3G14980	IDM1	938	133697	41	13	13.1	14.6
IPI00524605	AT5G59800	MBD7	366	35127	21	10	35	34.1
IPI00531355	AT1G54840	IDM2	305	39712	20	8	22.1	30.2

### Confirmation of protein-protein interactions in a IDM1 and IDM2 protein complex

**Fig 1:**
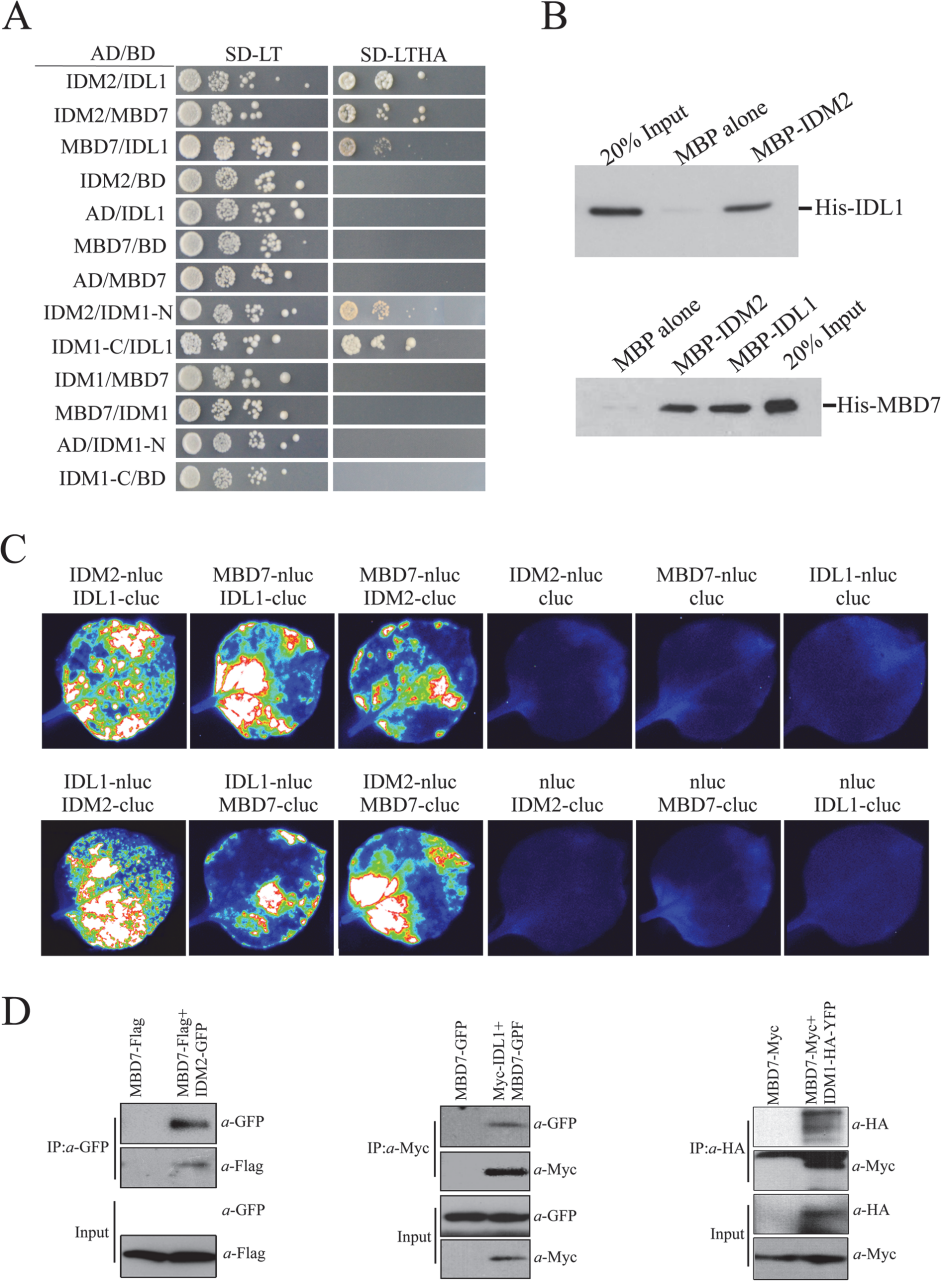
Protein-protein interactions between IDM1, IDM2, IDL1 and MBD7. (A) Yeast two-hybrid assays. Yeast cells carrying different fusion protein combinations are listed on the left. Yeast cells expressing the indicated proteins from the pGBK-T7 (BD) and pGAD-T7 (AD) vectors were plated onto medium lacking Leu and Trp (SD-LT) (left) or medium lacking Leu, Trp, Ade and His (SD-LTHA) (right). **(B)** Pull-down assays showing that IDM2, IDL1 and MBD7 interact with each other. **(C)** Split-luc assays showing that IDL1 and MBD7 can interact with IDM2 in *N*. *benthamiana* leaves. Three biological replicates were performed, and similar results were obtained. (D) Co-immunoprecipitation of MBD7 with IDM2 or IDL1 in tobacco leaves. MYC-tagged IDM2 and GFP-tagged IDM1 were transiently expressed in *N*. *benthamiana* leaves. Anti-GFP was used for immunoprecipitation (IP); anti-MYC and anti-GFP were used for immunoblotting; Input, total protein before immunoprecipitation. Transgenic plants expressing MBD7-Myc or IDM1-HA-YFP under their native promoters and their F1 offspring were used for co-IP.

The interaction of IDL1 and MBD7 with IDM1 and IDM2 was corroborated using the yeast two-hybrid assay (Fig 1A). Interestingly, IDL1 can interact with the C-terminal of IDM1 while IDM2 can interact with the N-terminal of IDM1 (Fig 1A). MBD7 also interacts with IDM2 and IDL1, but not with IDM1 (Fig 1A). The association between IDM2, IDL1, and MBD7 was also confirmed by a pull down assay (Fig 1B). To confirm these protein-protein interactions *in vivo*, we performed two assays. First, results from the split-LUC assay demonstrated that IDL1 interacts with IDM2, and that MBD7 interacts with both IDM2 and IDL1 (Fig 1C). Second, experiments using GFP-tagged IDM2 and Flag-tagged MBD7 transiently expressed in *Nicotiana benthamiana* leaves revealed that MBD7 co-IP’d with IDM2 from IDM2-GFP-expressing leaves, but not from leaves of control plants (Fig 1D). MBD7 also co-IP’d with IDL1 (Fig 1D). The interaction between MBD7 and IDM1 was also detected by co-IP in plants harboring both MBD7-Myc and IDM1-HA-YFP transgenes driven by their native promoters (Fig 1D). Taken together, our results suggest that IDM1, IDM2, IDL1 and MBD7 form a tight multiprotein complex *in vivo*.

### The *mbd7* mutation leads to DNA hypermethylation at specific loci

*IDL1* encodes an α-crystallin domain protein that belongs to the IDM2 protein family. Besides IDM2, the IDM2 protein family includes three IDM2-like proteins including IDL1 (At1g20870), IDL2 (At1g54850), and IDL3 (At1g76640) (S2 Fig). According to sequence alignment and phylogenetic analysis, IDL1 belongs to the same clade as IDM2 and shares maximal sequence similarity with IDM2 [21]. *MBD7* encodes a methyl-CpG-binding domain protein, which binds methylated CpG dinucleotides *in vitro* and localizes to highly CpG-methylated chromocenters in vivo *[17,18].*

**Fig 2:**
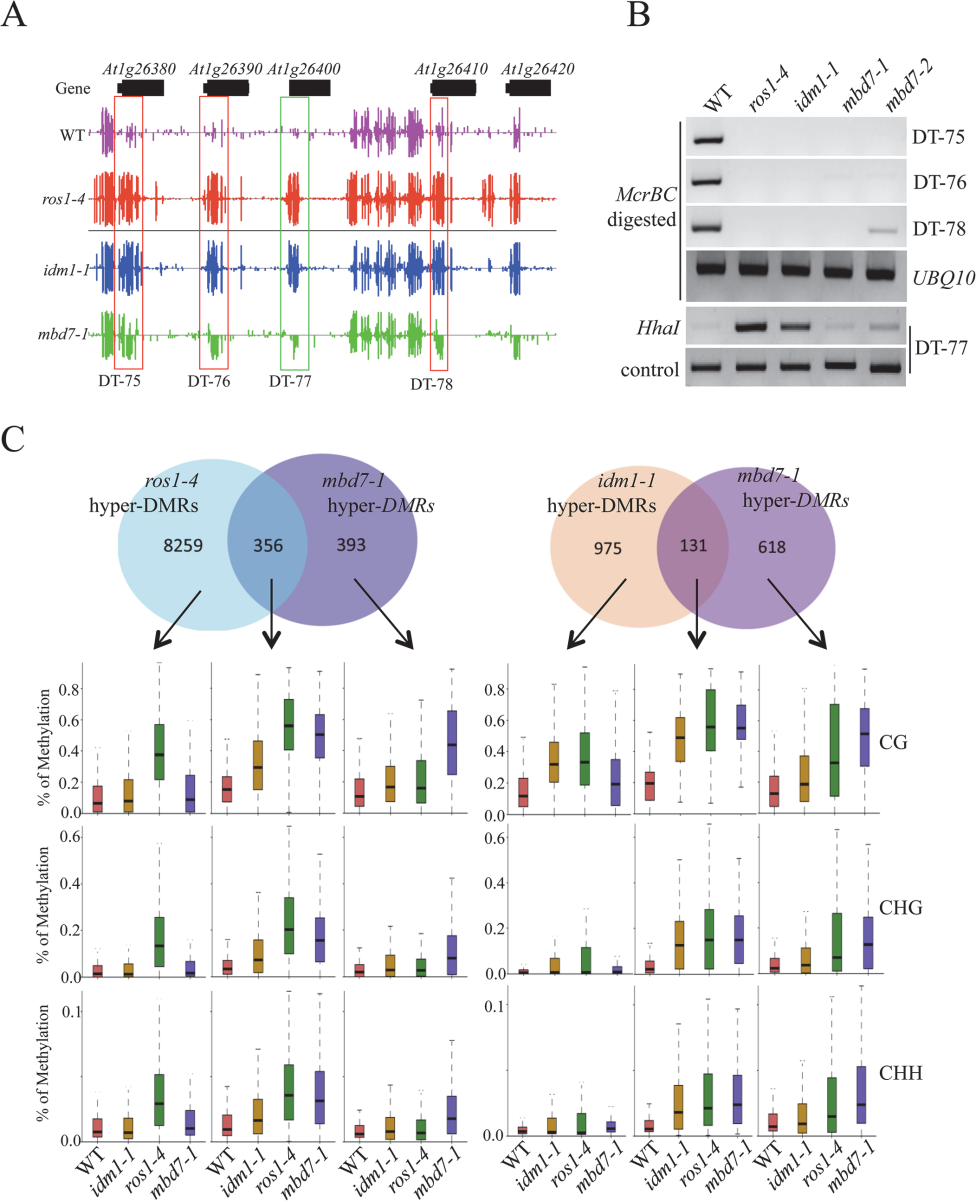
Methylome analysis of *mbd7-1* mutant. (A) Snapshot in the Integrated Genome Browser showing DNA methylation levels of the FAD-binding berberine gene family in WT, *idm1-1*, *ros1-4* and *mbd7-1*. Hypermethylated regions in *mbd7-1* were highlighted with red boxes. DT-77, which was hypermethylated in *idm1-1* or *ros1-4* but not in *mbd7-1*, was highlighted with green box. (B) Confirmation the whole genome bisulfite sequencing results by chop PCR. (C) Overlap of differentially methylated loci between *mbd7-1* and *ros1-4* and *idm1-1*. Boxplots represent methylation levels of each class of differentially methylated loci.

The tight association of IDL1 and MBD7 with IDM1 and IDM2 suggests that IDL1 and MBD7 may also be required for active DNA demethylation. In order to examine the possible role of MBD7 in active DNA demethylation, two T-DNA insertion lines were isolated and RT-PCR was done to confirm that they have a complete loss of mRNA expression (S3 Fig). Whole genome bisulfite sequencing was done using DNA purified from 12-day-old *mbd7-1* and wild type seedlings. The overall genome methylation level was slightly increased in *mbd7-1*mutant plants compared with wild type control (S4 and S5A Figs). Hundreds of differentially methylated regions (DMRs) were apparent in *mbd7-1* knockout plants, including 748 loci with DNA hypermethylation and 198 loci with DNA hypomethylation, respectively (S2 and S3 Tables). Six hypermethylated loci in *mbd7*, *idm1*, and *ros1* plants were selected for validation by PCR-based DNA methylation analysis, and all of them confirmed to be hypermethylated in *mbd7* mutants (Fig 2A and 2B and S5B and S5C Fig), as in *idm1-1* or *ros1-4*. However, the chop PCR marker locus DT-77, which was hypermethylated in *idm1* and *ros1* [10,11], was not hypermethylated in *mbd7* mutant plants (Fig 2B), suggesting not all IDM1 target loci were affected by MBD7 loss-of-function.

Same as the results in *idm1-1* and *ros1-4* mutants, the hypermethylated loci in *mbd7-1* mutants spread evenly across the five chromosomes and there was no enrichment at the chromocenters (S5D Fig), although MBD7 are mainly localized at highly methylated chromocenters in nuclei [18]. To determine whether the *MBD7* mutation affects DNA demethylation in gene body regions or in TEs, DNA regions were classified into gene body regions, intergenic regions, TEs out of gene regions, and TEs overlapping with gene regions. The ratios of DMRs in each class were calculated. Interestingly, we found the ratios of hypermethylated loci in each class of *mbd7-1* were similar to those of *ros1-4* (S5E Fig). Both of these two mutants have a small number of hyper DMRs distributed in gene body regions. We also examined the methylation levels of *ros1-4* and *idm1-1* in those regions that are hypermethylated in *mbd7-1* in CG, CHG, and CHH contexts and found most of them are hypermethylated to different degrees (S5F Fig). Approximately 18% and 48% of the 748 hyper-DMRs in *mbd7-1* were also hypermethylated in the *idm1-1* and *ros1-4* mutants, respectively (Fig 2C). We found the hyper-DMRs identified from *ros1-4* or *idm1-1* that overlap with *mbd7-1* mutant showed higher density of CG methylation compared with the non-overlapped loci (S5G Fig). This result suggests MBD7 dysfunction preferentially affects the genomic regions with high CG methylation density. The overlapped loci between *mbd7-1* and *ros1-4* were more frequently located in TE regions while the overlapped loci between *mbd7-1* and *idm1-1* were preferentially located in gene body regions (S5H and S5I Fig). The overlapped loci in these mutants are all hypermethylated in all sequence contexts (Fig 2C). The DNA methylation level at the *mbd7*-specific loci also appeared to be slightly increased in *idm1-1* and *ros1-4* (Fig 2C). However, the increases in *ros1-4* and *idm1-1* were not significant to make the loci counted as hyper-DMRs according to the parameters defined in this study, suggesting possible underestimation of the ratio of overlapping (Fig 2C). Taken together, our whole genome bisulfite sequencing results indicate that MBD7, IDM1 and ROS1 may function in the same pathway in active DNA demethylation at some loci.

### MBD7 prevents the silencing of a reporter gene and a subset of endogenous genes

**Fig 3:**
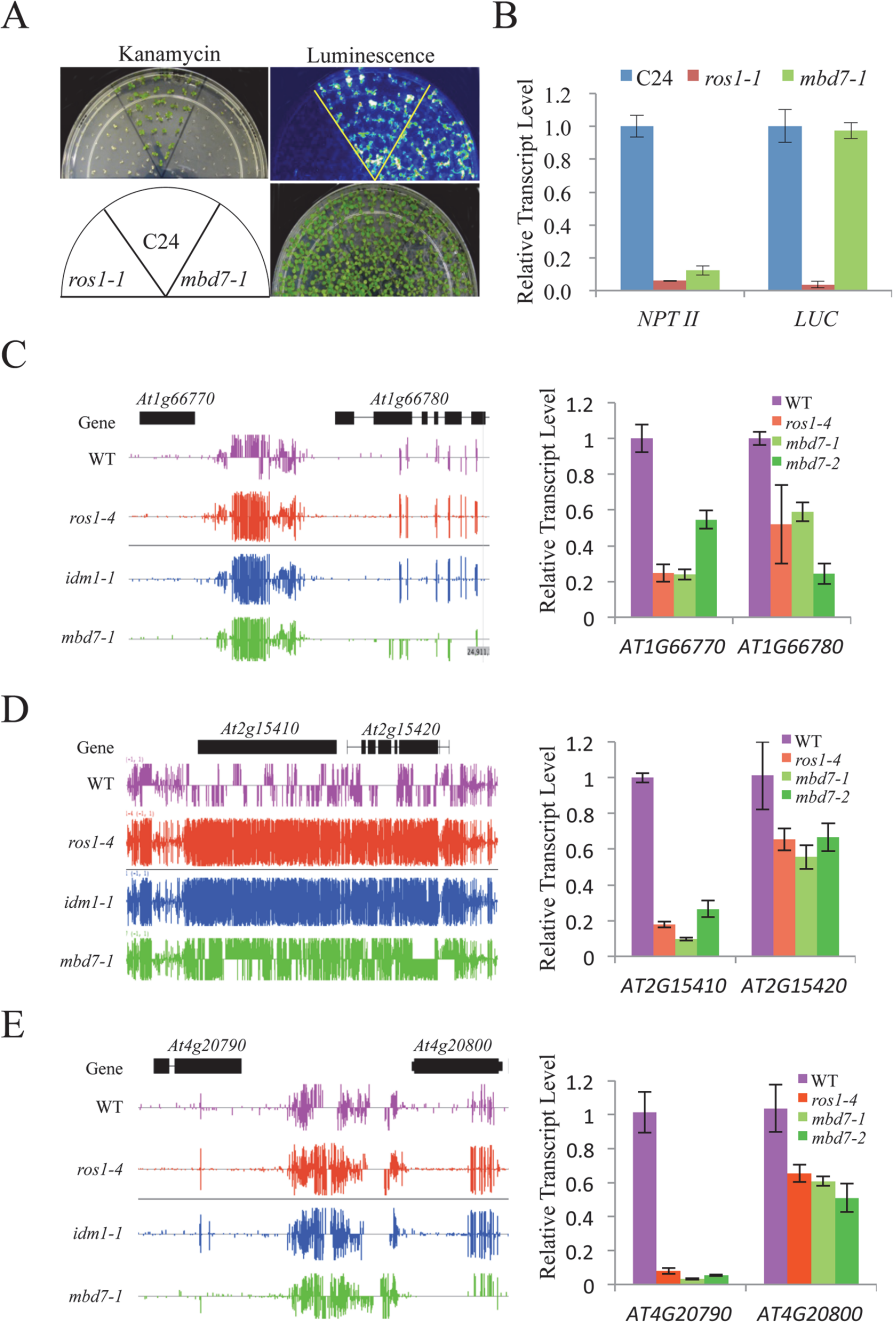
MBD7 prevents the silencing of a report gene and some endogenous genes. (**A**) The *mbd7-1* mutation causes the silencing of *35S-NPTII* but not the *RD29A-LUC* transgene. Seedlings grown in MS plates were imaged after cold treatment at 4°C for 24 h. For kanamycin resistance test, the seeds were planted on MS medium supplemented with 50 mg/L kanamycin and incubated for 2 weeks before being photographed. The reporter genes were introduced to *mbd7-1* mutant plants by crossing. (**B**) Real-time PCR analysis of the expression level of *LUC* and *NPTII* reporter genes in the different genotypes. (**C-E**) Effect of *mbd7* mutation on the expression of endogenous genes. Left column: Snapshot in the IGB showing DNA methylation levels in *ros1-4*, *idm1-1*, *mbd7-1* mutant plants and wild type control; right column: real-time PCR analysis of the expression level of the hypermethylated genes or nearby genes in different genotypes. *TUB8* was used as an internal control. Error bars represent standard error (*n* = 3).

Previous studies indicated that IDM1 and IDM2 were required for preventing certain transgenes and endogenous genes from transcriptional silencing [10,11,20,22]. To determine whether MBD7 functions in anti-silencing of transgenes, we introduced the *RD29A-LUC* and *35S-NPTII* into *mbd7-1* mutant plants by crossing the *mbd7-1* mutant with wild type C24 plants expressing the reporter gene. As shown in Fig 3A, the *mbd7-1* mutant exhibited a kanamycin sensitive growth phenotype but normal *LUC* expression. Real time PCR analysis also showed reduced expression of the *NPTII* transgene, but the expression level of *RD29A-LUC* in *mbd7-1* was comparable to that of the wild type control (Fig 3B). Thus, the *mbd7* mutation preferentially causes the silencing of *35S-NPTII* but not *RD29A-LUC*, consistent with the *idm1* or *idm2* mutation [20,22].

Changes in the DNA methylation level in or near a gene may modulate the expression of that gene. To determine whether DNA hypermethylation affects the expression of adjacent genes in *mbd7* mutant plants, we examined the expression levels of 14 genes that have detectable levels of transcript by real time PCR and show increased DNA methylation near or within the gene in *mbd7-1*, *idm1-1* and *ros1-4* plants (Fig 3C–3E and S6 Fig). Eleven of the tested genes showed a significant reduction in their transcript levels in these mutants (Fig 3C–3E and S6 Fig). Our results suggest that, like ROS1 and IDM1, MBD7 is critical for preventing the transcriptional silencing of some loci.

### MBD7 is important for the *in vivo* function of IDM1

**Fig 4:**
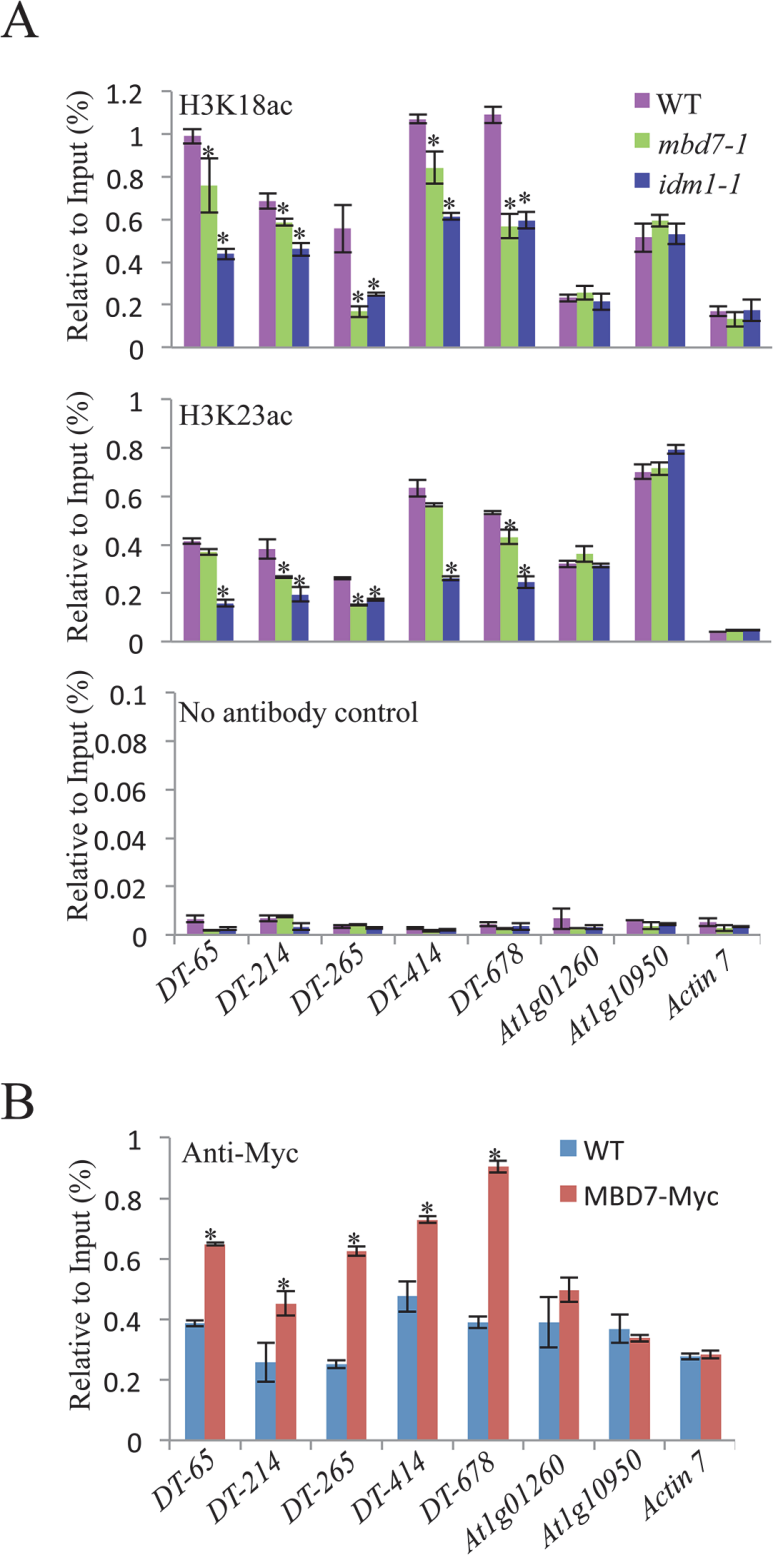
Histone acetylation marks and MBD7 association with chromatin. (A) H3K18 acetylation (ac) and H3K23ac levels at the hyper-DMR loci and control regions. ChIP was performed with antibodies against H3K18ac and H3K23ac. The ChIP signal was quantified relative to input DNA. The no-antibody precipitates served as negative control. Two biological replicates were performed, and very similar results were obtained. Standard errors were calculated from three technical repeats, *P < 0.05. (B) Association of MBD7 protein with hyper-DMR loci. ChIP was performed in WT and *MBD7-Myc* transgenic plants with antibody against Myc.

MBD7 can bind to methylated DNA through the methyl-CpG-binding domain *in vitro* [17], suggesting that MBD7 may recognize hypermethylated DNA regions and recruit IDM1 complex to the target loci. Previous data showed that IDM1 is critical for H3K18 and H3K23 acetylation *in vivo* [10]. Chromatin immunoprecipitation (ChIP) assays revealed that the acetylated histone H3K18ac and H3K23ac markers were reduced in *idm1-1* and *mbd7* mutant plants at hyper-DMRs, although the reduction in *mbd7-1* was not as dramatic as in *idm1-1* (Fig 4A). ChIP assays also indicated that MBD7 was enriched at all of the hyper-DMRs tested, but not the control regions that contain pretty high CpG DNA methylation in the gene body regions (like *At1g01260* and *At1g10950*) but did not show DNA hypermethylation in *mbd7-1* mutant compared with wild type (Fig 4B).

## Discussion

ROS1/DME family of 5mC DNA glycosylases initiated DNA demethylation is the major active DNA demethylation pathway in plants [23]. Unlike the well-established DNA methylation pathway, in which enzymes responsible for methylating DNA are guided to specific loci by base-pairing between small RNAs and the scaffold transcripts [1,24], how the DNA demethylation enzymes are recruited to specific genomic loci is poorly understood. Our findings demonstrate that, in addition to IDM1 and IDM2, MBD7 and IDL1 are required for ROS1-mediated active DNA demethylation and suppression of gene silencing at some loci in *Arabidopsis*.

DNA methylation is a conserved epigenetic marker that is important for TE and gene silencing, whereas DNA demethylation positively regulates gene expression. Some stress responsive genes are silenced by DNA methylation under normal conditions, but are induced by ROS1/DME mediated DNA demethylation under stressed conditions. The dynamic regulation of these genes is important for stress responses in *Arabidopsis* [25]. For example, the *ros1* and *rdd* (*ros1 dml2 dml3*) triple mutant shows increased susceptibility to the fungal pathogen *Fusarium oxysporum* [25]. In light of the important roles of MBD7, IDM1 and IDM2 in DNA demethylation, we speculate that this complex is important for activating stress responsive genes via recruiting ROS1 for active DNA demethylation.

IDM2 encodes an α-crystallin domain (ACD) protein that interact with IDM1 and is required for the full activity of IDM1 *in vivo* [11]. IDL1 is also an ACD protein and shares the maximal sequence identity with IDM2. Due to the lack of mutants for *IDL1* in the public database, we were unable to investigate the effects of *IDL1* mutation on DNA methylation level. IDL1 directly interacts with IDM1, IDM2, and MBD7. Our co-IP and MS/MS results suggested that equal amounts of IDM1, IDM2, IDL1, and MBD7 polypeptides are present in the IDM1 complex (Table 1), indicating that IDL1 may be as important as MBD7, IDM1 and IDM2 for active DNA demethylation and gene activation.

Our results also revealed that dysfunction of MBD7 causes DNA hypermethylation at hundreds of genomic loci and silencing of a reporter gene and lots of endogenous genes. Because MBD7 binds to the methylated DNA, the role of MBD7 in DNA demethylation may be facilitating the recruitment of IDM1 to the specific loci. Since the ratio of overlapped hyper-DMRs between *mbd7* and *idm1* was low, MBD7 may also recruit histone modification enzymes other than IDM1 to create chromatin environment favorable for active DNA demethylation. In addition to MBD7, MBD5 and MBD6 also bind to methylated DNA *in vitro* [18]. Interestingly, MBD6 was also identified during MS/MS analyses of IDM1- and IDL1-associated proteins (S7A Fig and S1 Table) and our yeast two-hybrid and split-LUC results confirmed that MBD6 can interact with IDM2 and IDL1 (S7B and S7C Fig). Chop-PCR results shown that the DNA methylation level of two loci were increased in *mbd6* and *idm1-1* mutants but not in *mbd7-1* mutant plants (S7D Fig), suggesting that MBD6 may also involved in recruitment IDM1 to specific DNA regions, promoting active DNA demethylation. This is consistent with the fact only a subset of genomic regions demethylated by ROS1 and other related 5mC DNA glycosylases were influenced in the *mbd7* knockout plants.

When this manuscript was under review, Lang et al. and Wang et al. reported that MBD7 and IDM3 were required for active DNA demethylation at some loci in *Arabidopsis* [26,27]. IDM3 is synonymous with IDL1. The identification of the same two proteins by three independent studies further underlines the importance of MBD7 and IDL1/IDM3 in the active DNA demethylation pathway. More importantly, in our study, we identified MBD7 and IDL1 from affinity purification and mass-spectrometric analysis. We characterized the IDM1-IDM2-IDL1-MBD7 complex and found the ratio of these four proteins should be 1:1:1:1 in the complex. Moreover, in addition toMBD7, MBD6 was found to be required for the recruitment of IDM1 to some specific loci in our study.

**Fig 5:**
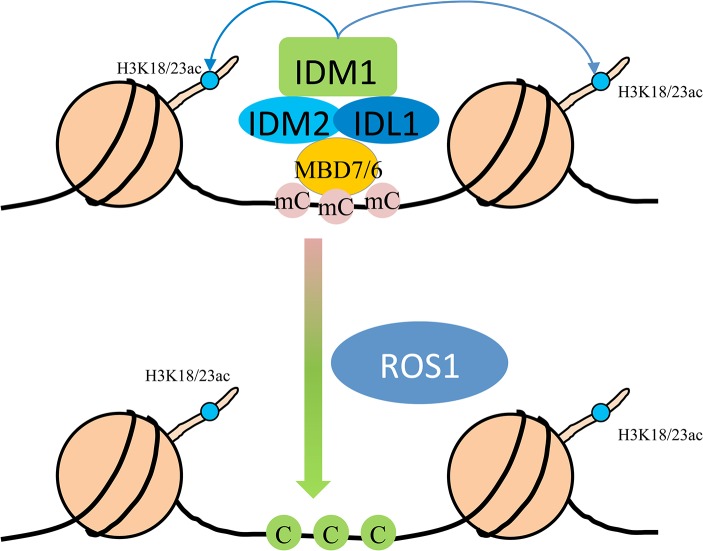
Working model for the IDM1-IDM2-IDL1-MBD7 complex functioning in ROS1 mediated active DNA demethylation at some loci in *Arabidopsis*. MBD7 forms a complex with IDM1, IDM2 and IDL1, and recognizes methylated DNA through methyl-CpG-binding domains. Then IDM1 is recruited to specific loci and acetylates histone H3 at K18 and K23, facilitating active DNA demethylation by ROS1.

In summary, we propose a model in which MBD7 or MBD6 binds to the DNA hypermethylated region and recruits the histone acetyltransferase complex (IDM1, IDM2 and IDL1) to specific loci. Histone modification (i.e., H3K18 and H3K23 acetylation) creates the favorable chromatin environment for targeting of ROS1 (and other members of the ROS1 subfamily of 5-methycytosine DNA glycosylases) to the loci for active DNA demethylation (Fig 5). In accordance with this model, MBD7 binds the target loci and H3K18ac and H3K23ac markers are reduced in the *mbd7* mutant plants, as in *idm1* mutants, at the target regions (Fig 4A). Not all the hypermethylated CG sites exhibited MBD7 accumulation (Fig 4B), suggesting that in addition to high CpG DNA methylation, other features such as heterochromatic histone marks may also contribute to MBD7 binding. MBD7 interacting proteins may also be involved in IDM1 targeting. As previous reported, IDM1 also contain a MBD domain at the N-terminal that can bind CG methylated nucleotide *in vitro* [10]. It seems that single MBD domain in IDM1 is not sufficient to recruit IDM1 to the methylated DNA and three additional MBD domains in MBD7 are required for the recognition of some of the IDM1 target loci. In addition, only some of the ROS1 target loci are affected in *idm1*, *idm2* and *mbd7*, indicating the existence of IDM1-independent mechanisms for recruiting the ROS1/DME glycosylases.

## Materials and Methods

### Plant materials and growth conditions

Two T-DNA insertion mutants of *MBD7*, *mbd7-1* and *mbd7-2*, were obtained from Arabidopsis Biological Resource Center. The *35S*::*6Myc-IDM2* transgenic plants were as described [11]. *Arabidopsis* seedlings were cultivated on Murashige-Skoog (MS) nutrient agar plates at 23°C with 16 h of light and 8 h of darkness for 12 days before DNA or RNA analysis.

### Cloning strategy

The *IDM1promoter*::*IDM1-3HA-YFP* construct was as described [10]. For the *35S*::*6Myc-IDL1* construct, full length of *IDL1* genomic DNA was amplified from wild type genomic DNA by PCR and sub-cloned into *pCAMBIA1307* (6Myc tag). For complementation of *mbd7* mutants, a 3.7-kb genomic DNA fragment containing the *MBD7* gene was amplified from Col-0 genomic DNA by PCR and cloned into the *pCAMBIA1305* vector for plant transformation. *Agrobacterium tumefacines* strain GV3101 carrying different constructs was used to transform mutant or wild type plants via the standard floral dipping method. Primary transformants were selected on MS plates containing hygromycin.

### Affinity purification and mass spectrometry

Approximately 5 g of flower tissue collected from IDM1-HA, IDM2-GFP and 6MYC-IDL1 transgenic plants was ground in liquid N_2_ with a mortar and pestle. The fine powder was suspended in 25 ml of lysis buffer (50 mM Tris, pH8.0, 230 mM NaCl, 5 mM MgCl_2_, 10% glycerol, 0.2% NP-40, 0.5 mM DTT, 1 mM PMSF, and 1 protease inhibitor cocktail tablet (Roche, 14696200)) and centrifuged for 15 min at 18,300 g at 4°C. The supernatant was incubated with 50 μl of anti-HA agarose beads (Sigma, A4865), GFP-Trap A beads (ChromoTek), or anti-c-Myc agarose beads (Sigma, A7470) for 5 h at 4°C. The beads were washed twice with 25 ml of lysis buffer and four times with 1 ml of lysis buffer. Proteins bound to HA beads were eluted with HA peptide (Genescript), whereas proteins bound to Myc beads were eluted with 0.1 M ammonium hydroxide (pH 11.5). The GFP-Trap A beads were directly boiled in SDS-sample buffer.

The mass spectrometric identification of purified proteins was done using the method described by Li et al. [28]. Briefly, purified proteins were separated by SDS-PAGE. Then the entire gel lane was cut into 6 equal portions and dehydrated. Proteins were digested in-gel with endoproteinase trypsin (0.5 ng/μL trypsin in 50 mM ammonium bicarbonate, pH 8.5). Extracted peptides were sequenced by LC-MS/MS on the Velos Pro Orbitrap Elite mass spectrometer (Thermo Scientific, USA) equipped with a nano-ESI source. The mass spectrometer was run in data-dependent mode with one MS scan in FT mode at a resolution of 120000. Ten CID (Collision Induced Dissociation) MS/MS scans were applied in the ion trap for each cycle [28]. Mascot server (Matrix Science Ltd., London, UK) and IPI (International Protein Index) *Arabidopsis* protein database were used as a searching platform.

### Yeast two-hybrid assay

The coding sequences of MBD7 and IDL1 were amplified by PCR. After validation of the sequences, the genes were subcloned into pGBK-T7 or pGAD-T7 (Clontech) to generate DNA binding or activation domain fusion constructs, respectively. For protein interaction analysis, two combinatory constructs were transformed simultaneously into the yeast strain AH109 (Clontech) and tested for Leu-, Trp-, and His- auxotrophy according to the manufacturer’s protocols.

### Split-LUC assay

The coding sequences of *MBD7 and IDL1* were amplified by PCR and subcloned into *pCAMBIA1300-NLUC* or *pCAMBIA1300-CLUC* to generate N-terminal or C-terminal luciferase-fusion constructs, respectively. For protein interaction analysis, two combinatory constructs were transformed simultaneously into *Nicotiana benthamiana* leaves. To prevent the silencing of those genes, a construct encoding virus p19 protein was transformed at the same time [29,30].

### Pull down assay

Pull down assays among IDM2, IDL2 and MBD7 were performed as described [31]. Briefly, Maltose binding protein (MBP) either alone or fused to IDM2 (MBP-IDM2) was expressed in E. coli and fixed to an amylose resin. His-tagged IDL1 purified from E.coli (His-IDL1) was incubated with either MBP-IDM2 or MBP bound to the resin. After washes, the proteins associated to the resin were separated by SDS-PAGE, transferred to a membrane, and immunoblotted with antibodies against His6- tag. To determin the interaction betweem MBD7 and IDM2 or IDL1, MBP alone or MBP-IDM2 or MBP-IDL1 was expressed in E. coli and fixed to an amylose resin. His-tagged MBD7 purified from E.coli (His-MBD7) was incubated with either MBP-IDM2 or MBP-IDL1 or MBP bound to the resin. After washes, the proteins associated to the resin were separated by SDS-PAGE, transferred to a membrane, and immunoblotted with antibodies against His6- tag.

### Agrobacterium tumefaciens-mediated transient co-expression

Over-expression binary constructs were transformed into *Agrobaterium tumefaciens* GV3101 and cultured overnight. After resuspending in 10 mM MgCl_2_, 150 μM acetosyringone and 10 mM MES (pH 5.7) at an OD_600_ of 0.6, and incubating for 3 h at room temperature in dark, the microbodies were syringed into leaves of *N*. *Benthamiana*. The infiltrated plants were grown in the greenhouse for 2–3 days.

### Co-immunoprecipitation analysis

For co-IP between MBD7 and IDM1, 1 g of flower tissue from MBD7-MYC and IDM1-HA-YFP and MBD7-MYC F1 hybrids were collected and ground in liquid N_2_. Lysis buffer (4 ml; 50 mM Tris pH8.0, 230 mM NaCl, 5 mM MgCl_2_, 10% glycerol, 0.2% NP-40, 0.5 mM DTT, 1 mM PMSF, 100 μM MG132 (Gene operation) and 1 protease inhibitor cocktail tablet (Roche, 14696200) was added and mixed carefully. After centrifugation for 15 min at 18,200 g and 4°C, the supernatants were incubated with 8 μL of anti-HA agarose (Sigma, A4865) overnight at 4°C while rotating. After washing the beads 5 times with 1 ml lysis buffer for 5 min each, the beads were resuspended in 60 μL SDS sample buffer and boiled for 5 min. Input and eluted proteins (20 μL and 15 μL, respectively) were resolved by 10% SDS-PAGE and electroblotted onto PVDF membranes for Western blotting. The primary antibodies against HA and MYC were diluted at 1:2000 and the second antibody goat anti-mouse IgG (Cwbio) was diluted at 1:5000.

For the co-IP between MBD7 and IDM2, MBD7 and IDL1, 0.3 g of co-transformed *N*. *Benthamiana* leaves were ground in liquid N_2_ and incubated in l ml lysis buffer without MG132. After centrifugation, 5 μL GFP-Trap A (ChromoTek) or anti-c-Myc agarose (Sigma, A 7470) were added and incubated for 4 h. The beads were washed and then boiled in 50 μL SDS sample buffer. About 20 μL of input and 10 μl of eluate were analyzed by SDS-PAGE and Western blotting. The primary antibody against GFP (EASYBIO), Flag (Sigma, F1804) and MYC (Cwbio) were diluted at 1:2000 and the second antibody goat anti-mouse IgG or goat anti-rabbit IgG (Cwbio) were diluted at 1:5000.

### Whole-genome bisulfite sequencing and data analysis

DNA was extracted from 2 g of 12-d-old seedlings using the DNeasy Plant Mini Kit (Qiagen) and sent to the Center for High throughput sequencing (Biodynamic Optical Imaging Center, Peking University) for bisulfite treatment, library preparation, and sequencing. For data analysis, adapter, and low-quality sequences (q < 20) were trimmed and clean reads were mapped to *Arabidopsis thaliana* TAIR 10 (10th release of the *Arabidopsis* genome sequence from the Arabidopsis Information Resource) genome using Bisulfite Sequence Mapping Program allowing two mismatches. Identification of differentially methylated regions (DMRs) was conducted according to [32]. Heat maps were drawn according to [22].

### Chop-PCR

Genomic DNA (100 ng) was cleaved with the methylation-sensitive restriction enzymes *HhaI* or the methylation-dependent restriction enzyme *McrBC*, and the products subjected to PCR using the loci-specific primers (S4 Table).

### Real-time RT-PCR

Total RNA was extracted from 12-d-old seedlings in plates using the RNeasy plant mini kit (Qiagen), and contaminating DNA removed with RNase-free DNase (Qiagen). About 2 μg mRNA was used for first-strand cDNA synthesis with the Super script^TM^ III First-Strand Synthesis System (Invitrogen) for RT-PCR following the manufacturer’s instructions. The cDNA reaction mixture was then diluted five times, and 1 μl used as template in a 20 μl PCR reaction with iQ SYBR Green Supermix (Bio-Rad).

### ChIP assays

Chromatin immunoprecipitation (ChIP) assays were performed according to a published protocol [33]. The following antibodies were used for ChIP assays: anit-H3K18ac (ab1191, Abcam), anti-H3K23ac (07–355, Millipore), and anti-MYC (M4439, Sigma). ChIP products were eluted into 50 μl of TE buffer, and a 2 μl aliquot was used for each qPCR reaction.

### Accession numbers

We used whole genome bisulfite sequencing data to analyze the genome-wide methylation status in WT and *mbd7-1* mutant plants. The dataset was deposited at NCBI (Col-0:SRX747290, *mbd7-1*:SRX833671). The *ros1-4* and *idm1-1* whole genome bisulfite sequencing data were from GEO accession GSE33071 [10].

## Supporting Information

S1 Fig Complementation of *idm1-3* mutant with *IDM1-3HA-YFP*. (A) The *IDM1-3HA-YFP* transgene restored the kanamycin resistance in *idm1-3*. For kanamycin resistance test, the seeds were planted on MS medium supplemented with 50 mg/L kanamycin and incubated for 2 weeks before being photographed. (B) Real-time PCR analysis of the expression level of *NPT II* reporter gene in the different genotypes. (C) The IDM1-3HA-YFP protein levels in the transgenic line detected by Western blot. IDM1-HA-YFP was immunoprecipitated and detected by the anti-HA antibody.
(PDF)

S2 Fig Clustal analysis of the phylogenetic relationship for the IDM2 family. The phylogenetic tree was generated based on the sequence comparison of the α-crystallin domain (ACDs) only and using the method described by Scharf et al., 2001[21]. Amino acid sequences from At1g76440 (37–135), At1g54840 (93–192), At1g54840 (239–338), At1g20870 (360–459), At3g12570 (365–469), At2g37570 (364–468) and At5g02480 (373–477) were used to do the multiple sequence alignment using DNAMAN software.
(PDF)

S3 Fig Characterization of the *mbd7* and *mbd6* T-DNA insertion mutants. (A) Schematic diagram showing the positions of the T-DNA insertions at the *MBD7* and *MBD6* loci. Black rectangles represent exons. (B) Domain structure for the MBD7 and MBD6 proteins. Yellow boxes represent methyl-CpG-binding domains. (C-D) RT-PCR analysis of *MBD7* and *MBD6* transcript levels in wild type and mutant plants. *TUB8* served as a control. The positions of RT-PCR primers were indicated in S3A Fig.
(PDF)

S4 Fig Density of DNA methylation in *idm1-1*, *mbd7-1* and *ros1-4* mutants as compared to wild type plants. The densities of methylcytosines of each sequence context (CG, CHG, and CHH) across each chromosome in 50 kb segments are shown.
(PDF)

S5 Fig DNA methylome analysis by whole genome bisulfite sequencing in wild-type, *mbd7-1*, *idm1-1* and *ros1-4* mutant plants. (A) Average methylation levels in gene and TE bodies. Genes or TEs were aligned from 1kb upstream of transcription start sites to 1kb downstream of transcription termination sites. (B) Snapshot in the Integrated Genome Browser showing DNA methylation levels of the cysteine/histidine-rich C1 domain gene family. (C) Confirmation the whole genome bisulfite sequencing results by chop PCR. (**D**) Distribution of hypermethylated loci on the five chromosomes in *mbd7-1*, *idm1-1* and *ros1-4* mutants. (**E**) Composition of the hypermethylated loci in *mbd7-1*, *idm1-1* and *ros1-4* mutants. (F) Heat map showing the methylation levels of *ros1-4* and *idm1-1* in those regions that are hypermethylated DMRs in *mbd7-1* in three contexts. Light yellow indicates low methylation and black indicates high methylation. (**G**) Box plots shown CG DNA methylation density (Y-axis) in different groups of DMRs (X-axis). (**H-I**) Composition of hyper-DMRs in different groups.
(PDF)

S6 Fig Examples of whole genome bisulfite sequencing data showing DNA hypermethylation in *mbd7-1*, *idm1-1* and *ros1-4* mutants and the effects of the mutations on expression of the hypermethylated genes or nearby genes. (A-D) Effect of *mbd7* mutation on the expression of endogenous genes. Left panel: Snapshot in the Integrated Genome Browser showing DNA methylation levels in the mutants. Right panel: Gene expression level in the mutants determined by real time PCR. *TUB8* was used as an internal control. Error bars represent standard error (n = 3). (E) Effect of *mbd7* mutation on the expression of *At3g62470*, *At3g62475*, *At3g62480* and *At3g62490*. Upper panel: Snapshot in the Integrated Genome Browser showing DNA methylation levels in the mutants. Lower panel: Gene expression level in the mutants determined by real time PCR. *TUB8* was used as an internal control. Error bars represent standard error (n = 3).
(PDF)

S7 Fig Identification of MBD6 by IDM1 or IDL1 purification and confirm the protein-protein interaction. (A) Mass-spectrometric analysis of IDM1 and IDL1 co-purifying proteins. (B) Split-luc assays showing that MBD6 can interact with IDM2 and IDL1 in *N*. *benthamiana* leaves. Three biological replicates were performed, and similar results were obtained. (C) Yeast two-hybrid assays. (D) Chop-PCR assay determine the DNA methylation level. Genomic DNA from wild type and mutant plants were digested with McrBC, a DNA methylation-dependent restriction enzyme.
(PDF)

S8 Fig Full western blot scans related to Fig 1D. (PDF)

S1 Table List of all identified peptides from mass-spectrometric analysis. (XLS)

S2 Table Hyper-DMRs in *mbd7-1*. (XLSX)

S3 Table Hypo-DMRs in *mbd7-1*. (XLSX)

S4 Table Primers used in this study. (DOCX)
