# Correction: Regulation of Active DNA Demethylation by a Methyl-CpG-Binding Domain Protein in *Arabidopsis thaliana*

**DOI:** 10.1371/journal.pgen.1005380

**Published:** 2015-07-30

**Authors:** Qi Li, Xiaokang Wang, Han Sun, Jun Zeng, Zhendong Cao, Yan Li, Weiqiang Qian

The second panel from the bottom of the left side of Fig 1D, is missing. The authors have provided a corrected version here.

**Fig 1:**
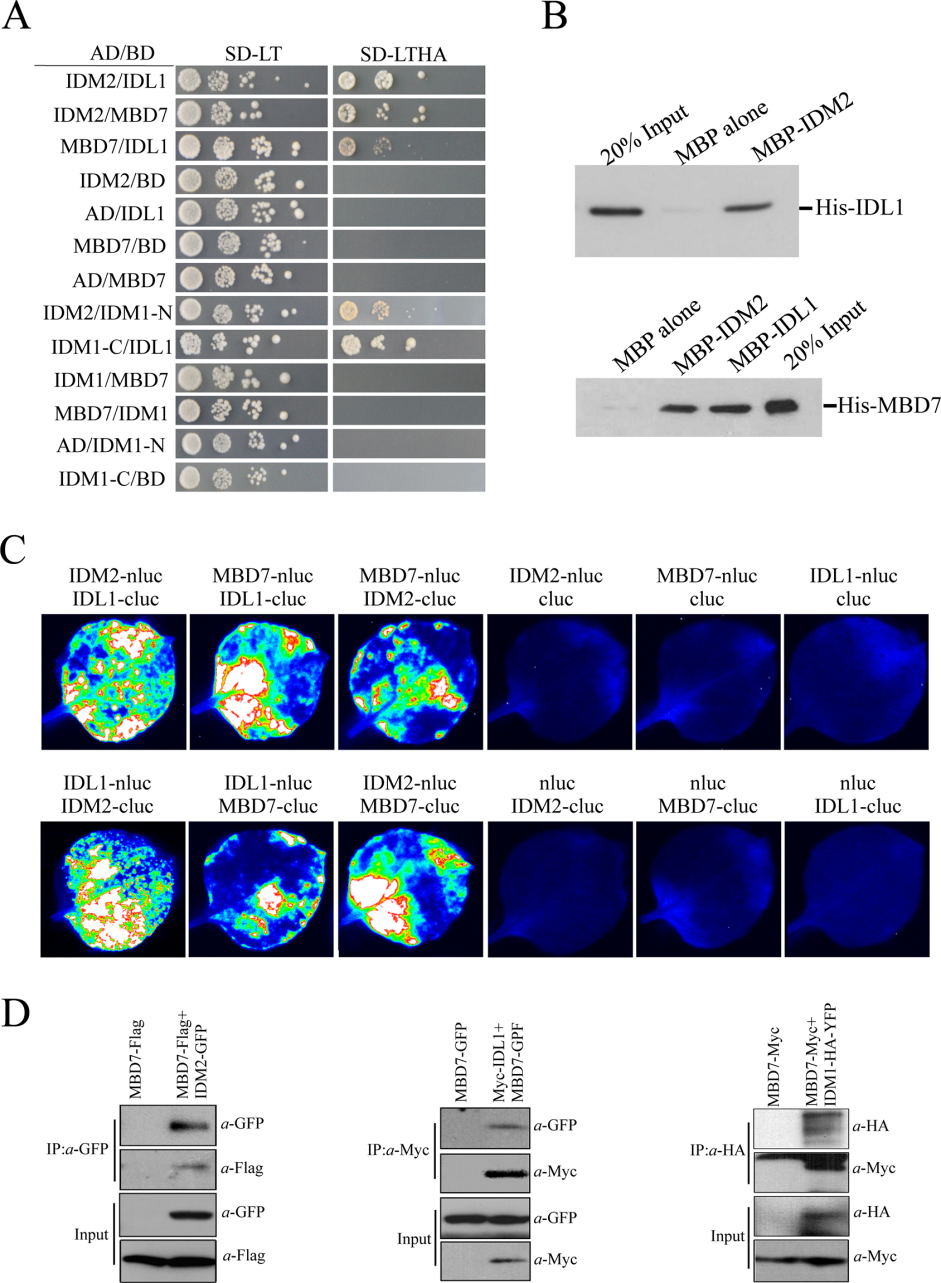
Protein-protein interactions between IDM1, IDM2, IDL1 and MBD7. (A) Yeast two-hybrid assays. Yeast cells carrying different fusion protein combinations are listed on the left. Yeast cells expressing the indicated proteins from the pGBK-T7 (BD) and pGAD-T7 (AD) vectors were plated onto medium lacking Leu and Trp (SD-LT) (left) or medium lacking Leu, Trp, Ade and His (SD-LTHA) (right). **(B)** Pull-down assays showing that IDM2, IDL1 and MBD7 interact with each other. **(C)** Split-luc assays showing that IDL1 and MBD7 can interact with IDM2 in *N*. *benthamiana*leaves. Three biological replicates were performed, and similar results were obtained. (D) Co-immunoprecipitation of MBD7 with IDM2 or IDL1 in tobacco leaves. MYC-tagged IDM2 and GFP-tagged IDM1 were transiently expressed in *N*. *benthamiana* leaves. Anti-GFP was used for immunoprecipitation (IP); anti-MYC and anti-GFP were used for immunoblotting; Input, total protein before immunoprecipitation. Transgenic plants expressing MBD7-Myc or IDM1-HA-YFP under their native promoters and their F1 offspring were used for co-IP.
