# Supplementary figures and images for: Regulation of Active DNA Demethylation by a Methyl-CpG-Binding Domain Protein in Arabidopsis thaliana
Source: PLoS Genet. 2015 May 1;11(5):e1005210. doi: 10.1371/journal.pgen.1005210 (PMC4416881; doi:10.1371/journal.pgen.1005210)

A

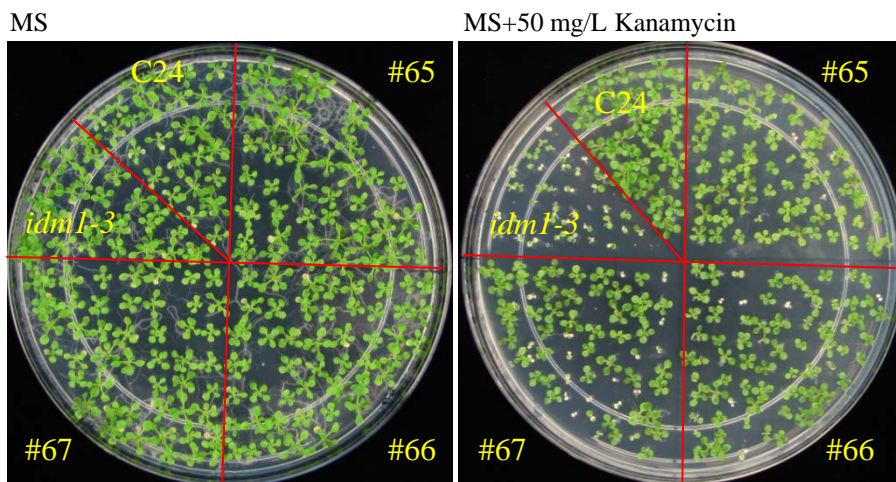

B

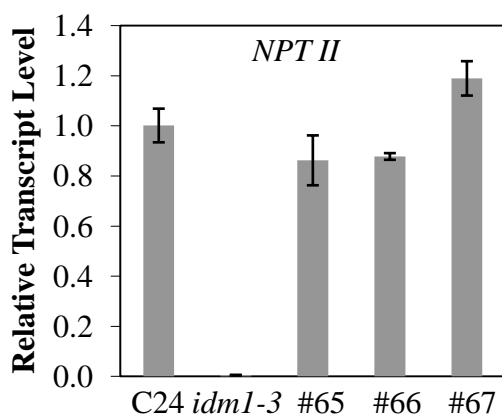

C

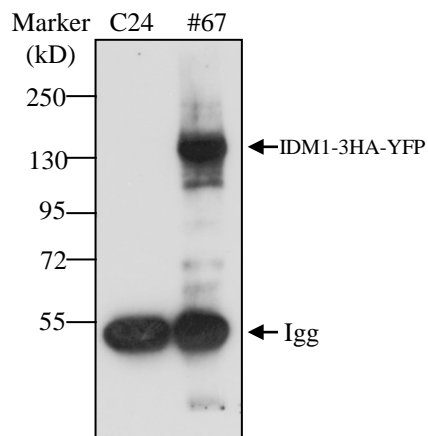

Figure S1

Supplement: S1 Fig — (A) The IDM1-3HA-YFP transgene restored the kanamycin resistance in idm1-3. For kanamycin resistance test, the seeds were planted on MS medium supplemented with 50 mg/L kanamycin and incubated for 2 weeks before being photographed. (B) Real-time PCR analysis of the expression level of NPT II reporter gene in the different genotypes. (C) The IDM1-3HA-YFP protein levels in the transgenic line detected by Western blot. IDM1-HA-YFP was immunoprecipitated and detected by the anti-HA antibody. (PDF) [file pgen.1005210.s001.pdf]

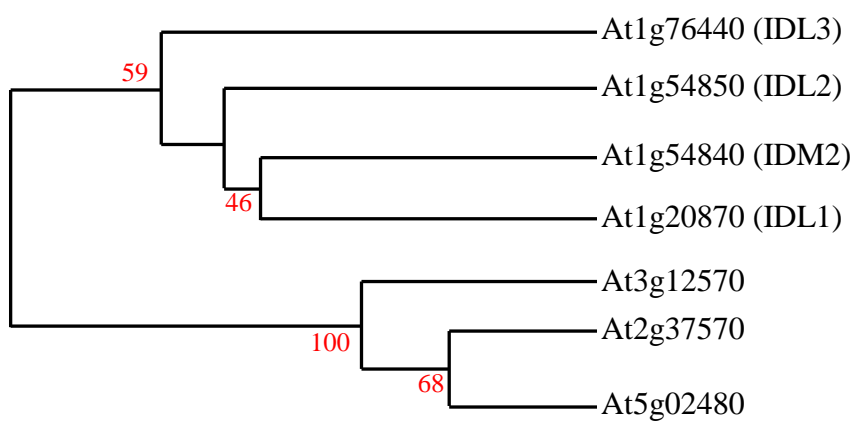

**Figure S2**

Supplement: S2 Fig — The phylogenetic tree was generated based on the sequence comparison of the α-crystallin domain (ACDs) only and using the method described by Scharf et al., 2001[21]. Amino acid sequences from At1g76440 (37–135), At1g54840 (93–192), At1g54840 (239–338), At1g20870 (360–459), At3g12570 (365–469), At2g37570 (364–468) and At5g02480 (373–477) were used to do the multiple sequence alignment using DNAMAN software. (PDF) [file pgen.1005210.s002.pdf]

A

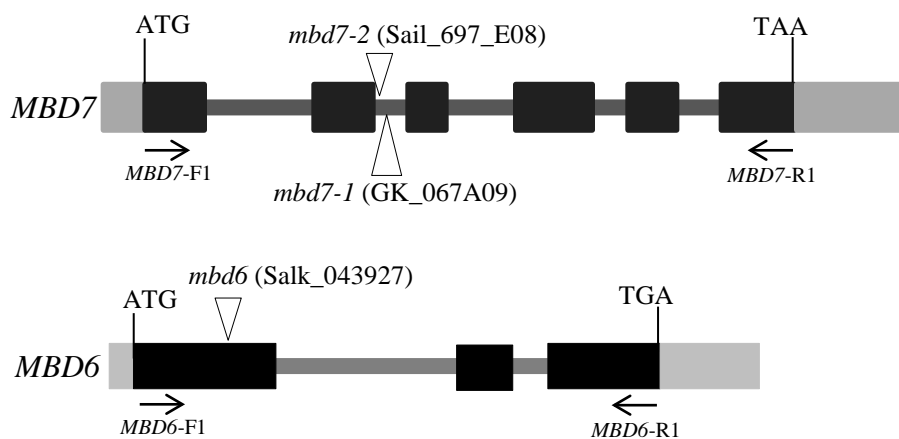

B

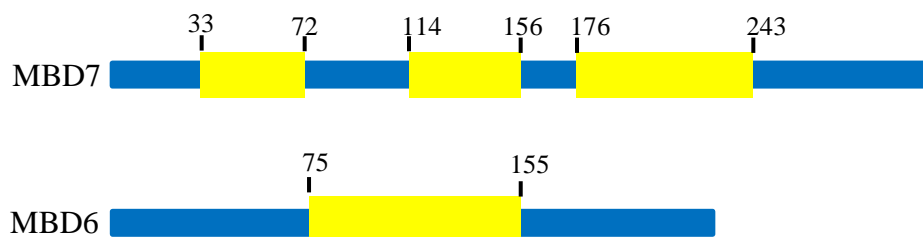

C

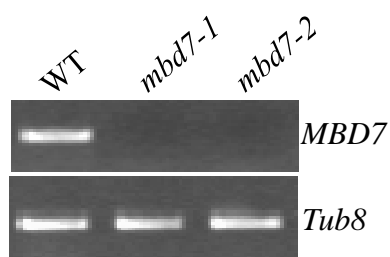

D

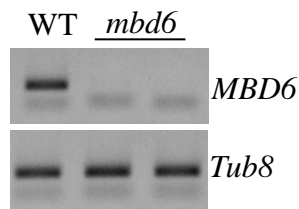

Figure S3

Supplement: S3 Fig — (A) Schematic diagram showing the positions of the T-DNA insertions at the MBD7 and MBD6 loci. Black rectangles represent exons. (B) Domain structure for the MBD7 and MBD6 proteins. Yellow boxes represent methyl-CpG-binding domains. (C-D) RT-PCR analysis of MBD7 and MBD6 transcript levels in wild type and mutant plants. TUB8 served as a control. The positions of RT-PCR primers were indicated in S3A Fig. (PDF) [file pgen.1005210.s003.pdf]

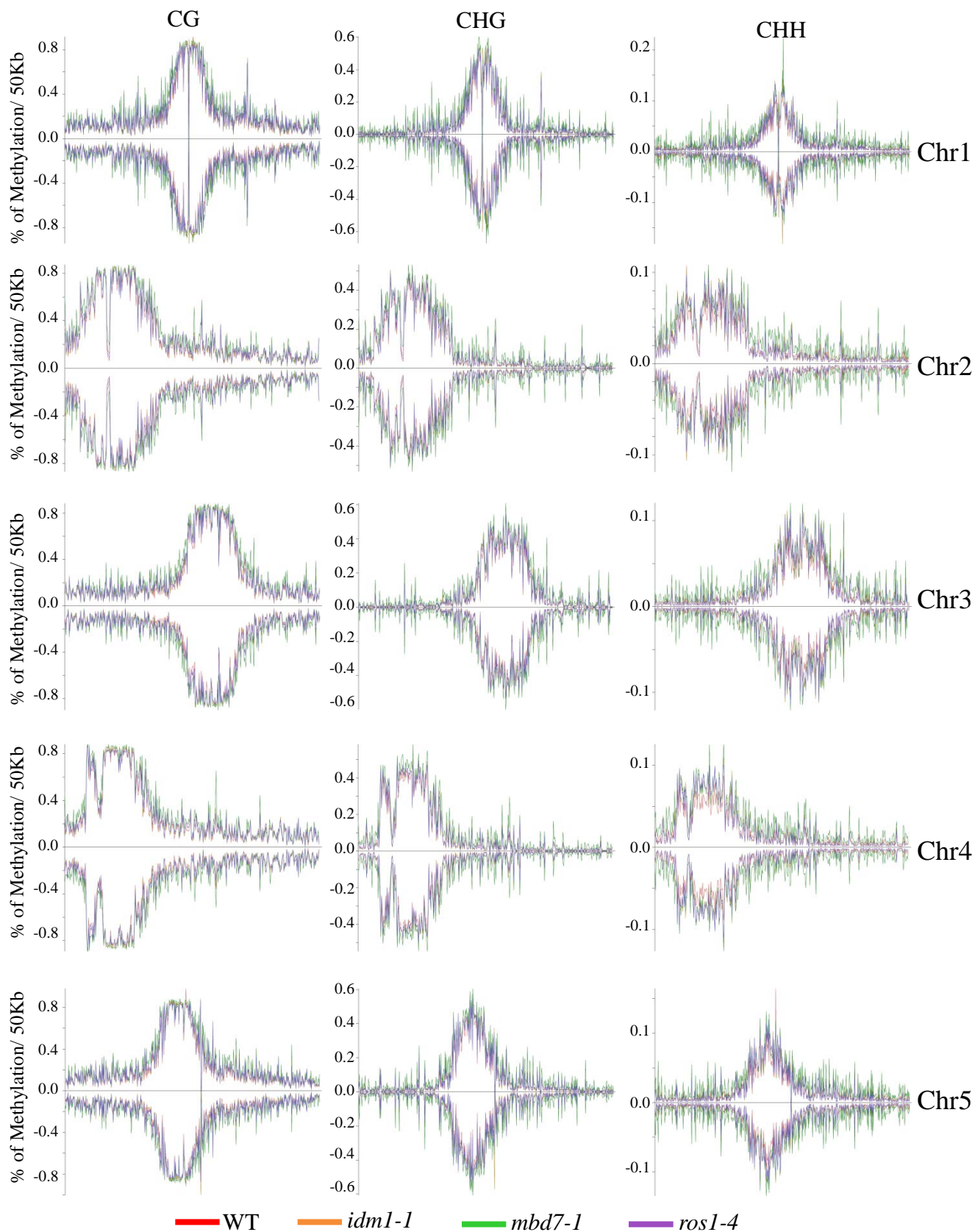

**Figure S4**

Supplement: S4 Fig — The densities of methylcytosines of each sequence context (CG, CHG, and CHH) across each chromosome in 50 kb segments are shown. (PDF) [file pgen.1005210.s004.pdf]

A

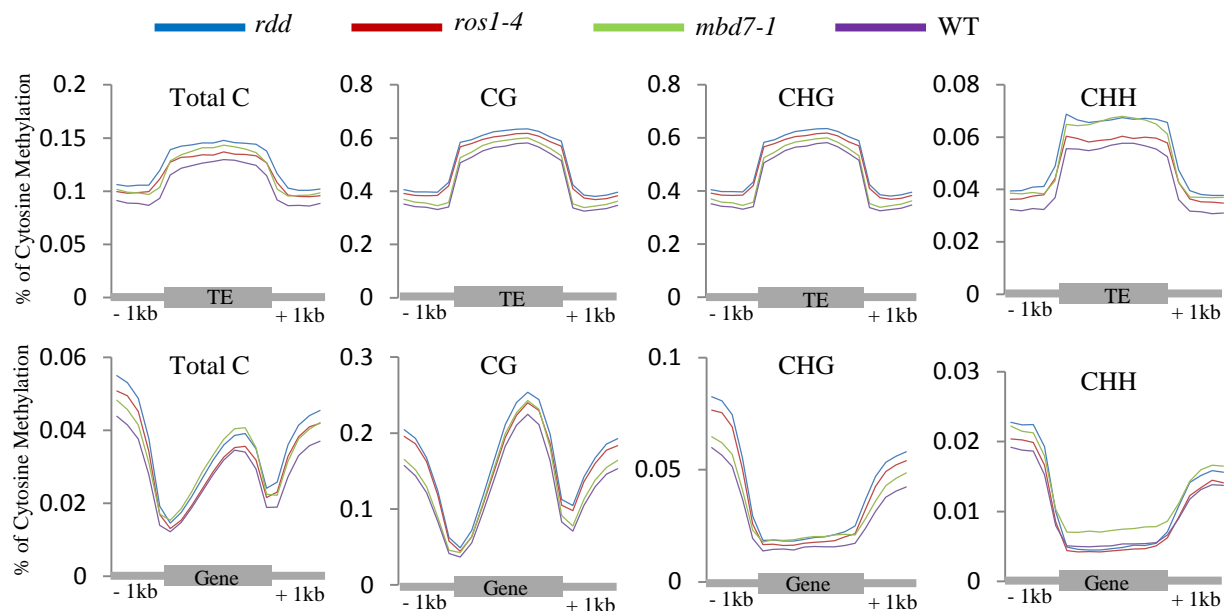

B

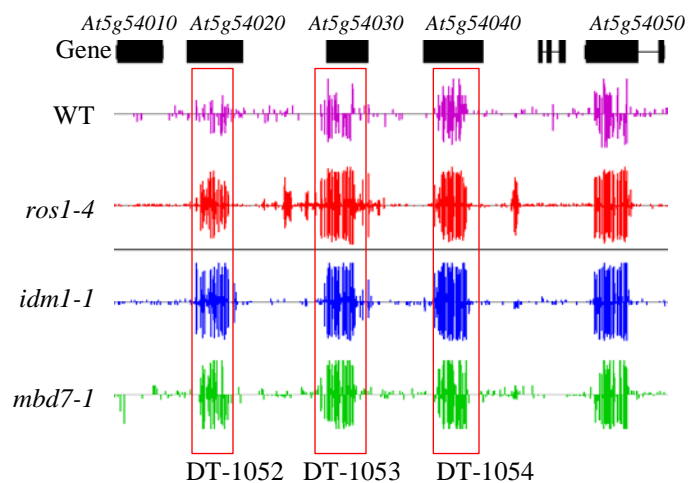

C

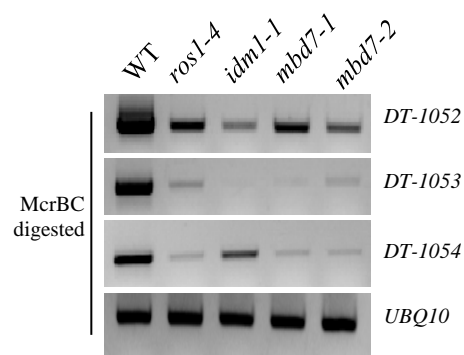

Figure S5

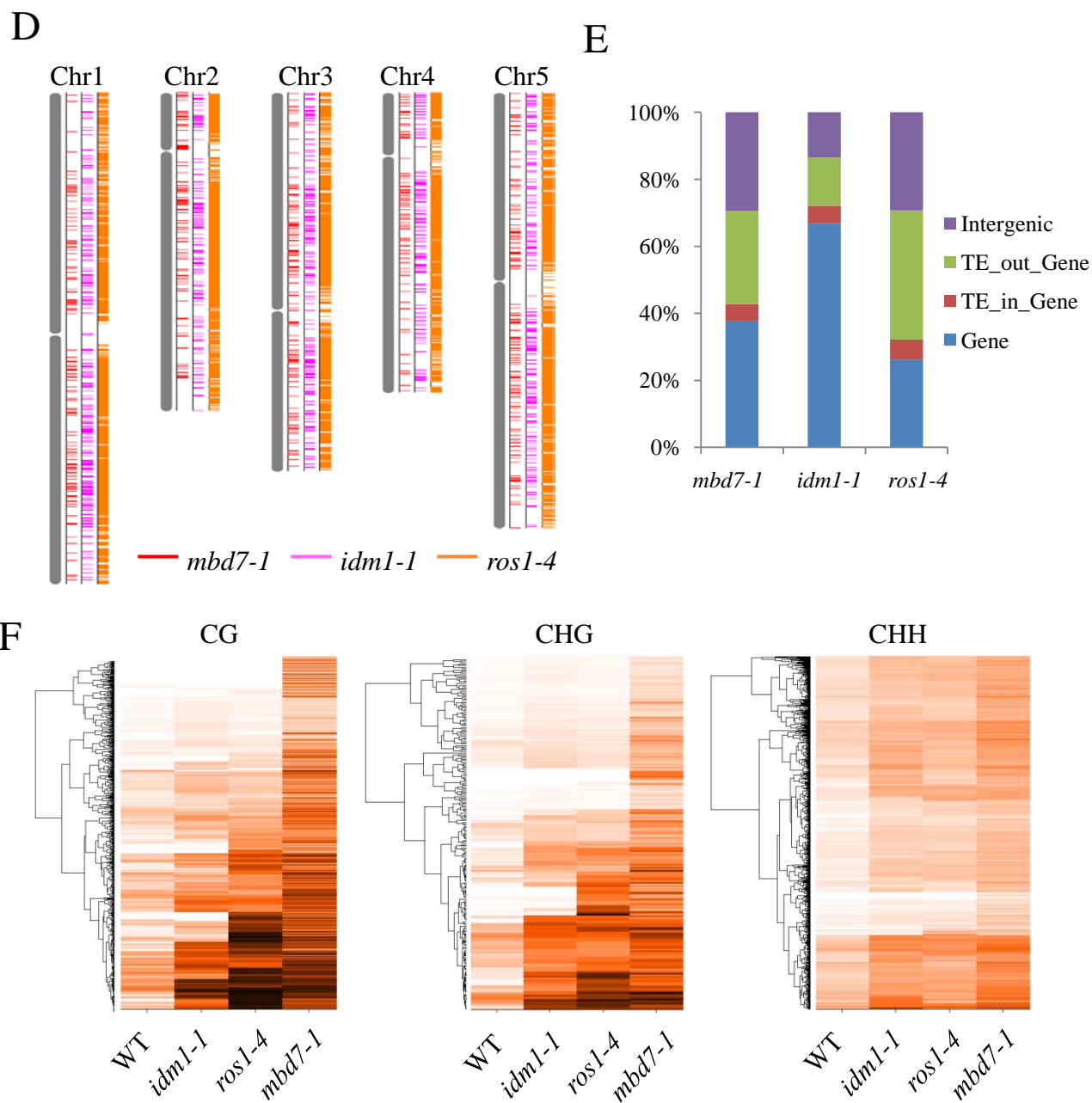

Figure S5 continued

G

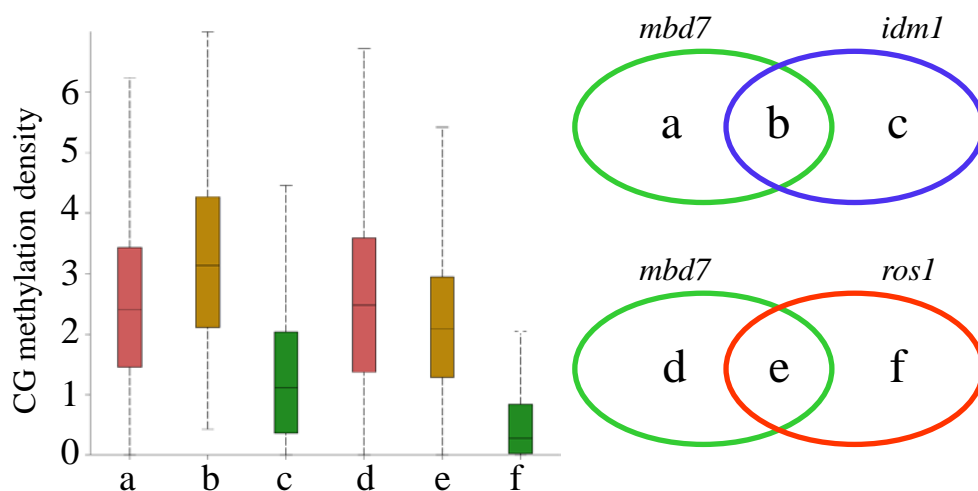

H

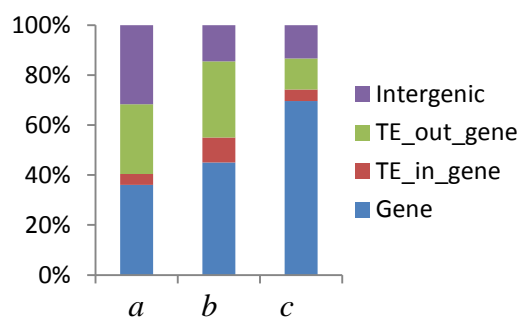

I

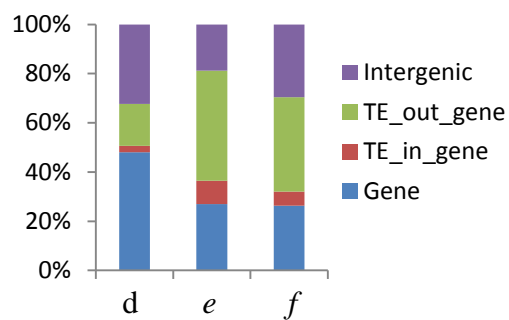

Figure S5 continued

Supplement: S5 Fig — (A) Average methylation levels in gene and TE bodies. Genes or TEs were aligned from 1kb upstream of transcription start sites to 1kb downstream of transcription termination sites. (B) Snapshot in the Integrated Genome Browser showing DNA methylation levels of the cysteine/histidine-rich C1 domain gene family. (C) Confirmation the whole genome bisulfite sequencing results by chop PCR. (D) Distribution of hypermethylated loci on the five chromosomes in mbd7-1, idm1-1 and ros1-4 mutants. (E) Composition of the hypermethylated loci in mbd7-1, idm1-1 and ros1-4 mutants. (F) Heat map showing the methylation levels of ros1-4 and idm1-1 in those regions that are hypermethylated DMRs in mbd7-1 in three contexts. Light yellow indicates low methylation and black indicates high methylation. (G) Box plots shown CG DNA methylation density (Y-axis) in different groups of DMRs (X-axis). (H-I) Composition of hyper-DMRs in different groups. (PDF) [file pgen.1005210.s005.pdf]

A

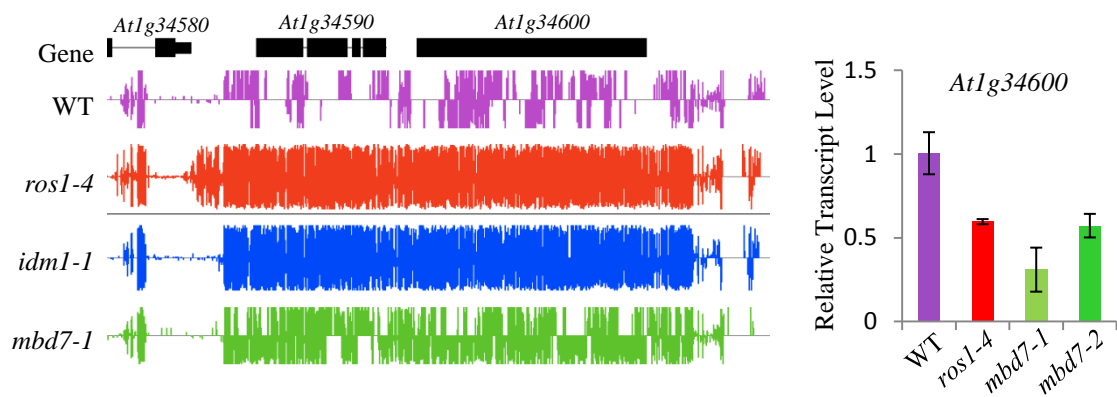

B

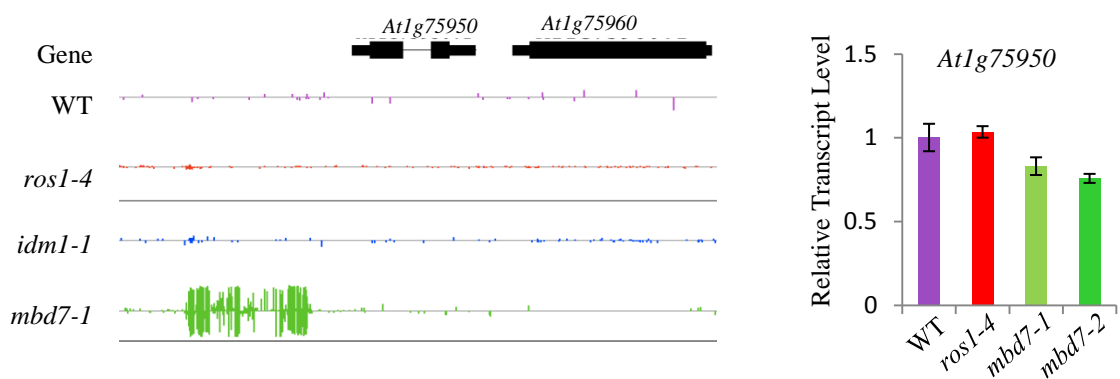

C

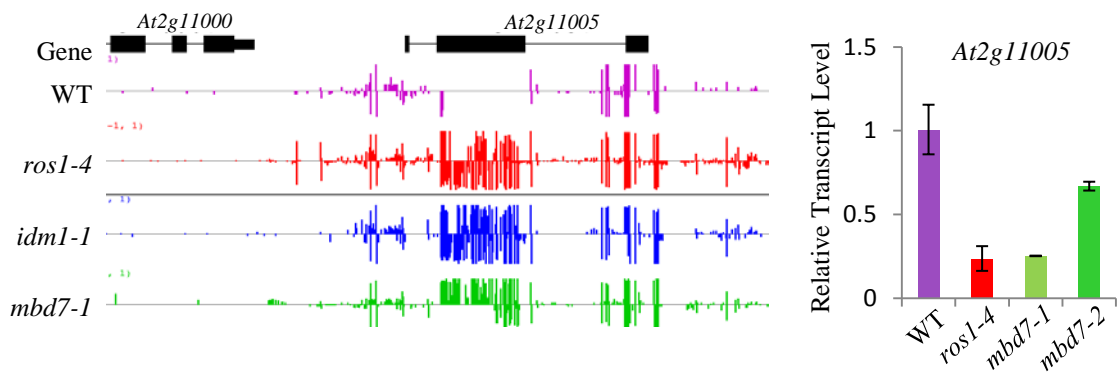

Figure S6

D

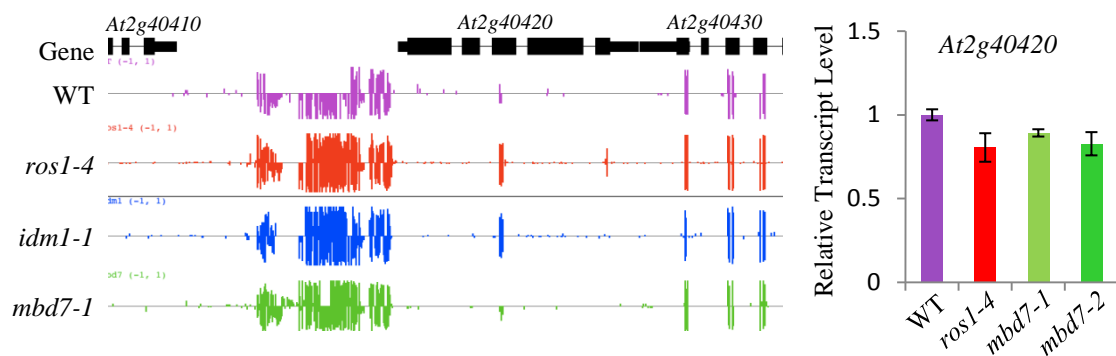

E

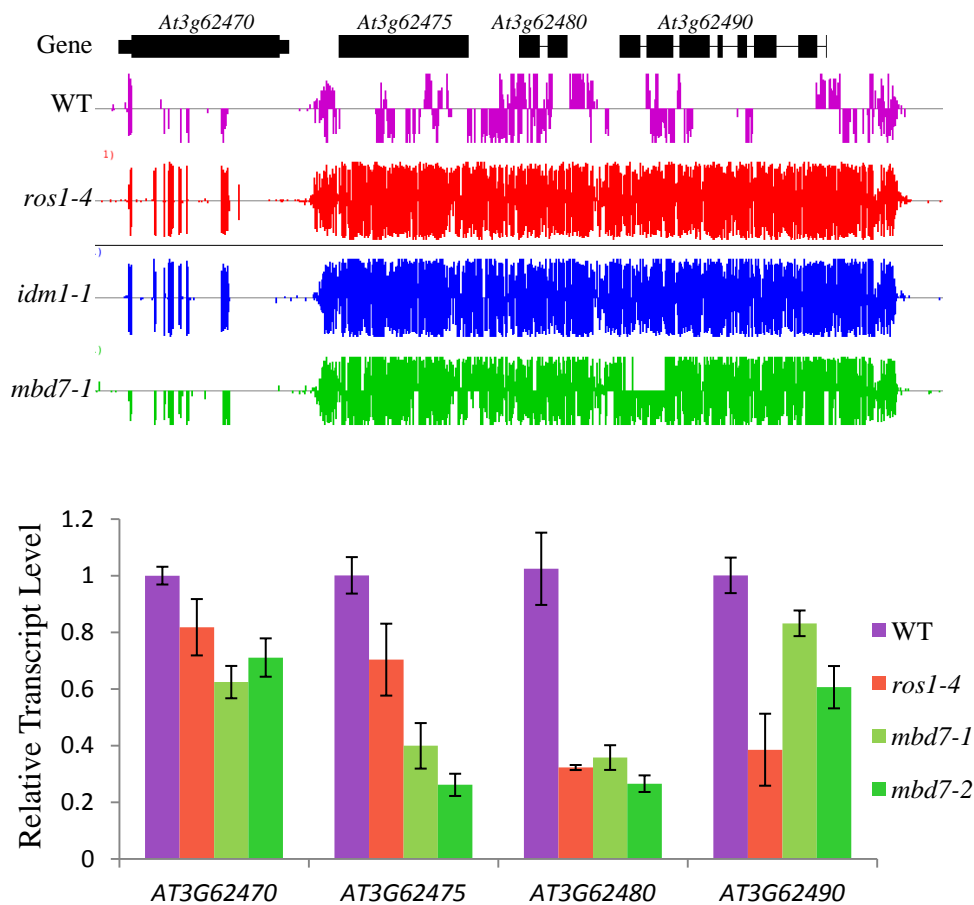

Figure S6 continued

Supplement: S6 Fig — (A-D) Effect of mbd7 mutation on the expression of endogenous genes. Left panel: Snapshot in the Integrated Genome Browser showing DNA methylation levels in the mutants. Right panel: Gene expression level in the mutants determined by real time PCR. TUB8 was used as an internal control. Error bars represent standard error (n = 3). (E) Effect of mbd7 mutation on the expression of At3g62470, At3g62475, At3g62480 and At3g62490. Upper panel: Snapshot in the Integrated Genome Browser showing DNA methylation levels in the mutants. Lower panel: Gene expression level in the mutants determined by real time PCR. TUB8 was used as an internal control. Error bars represent standard error (n = 3). (PDF) [file pgen.1005210.s006.pdf]

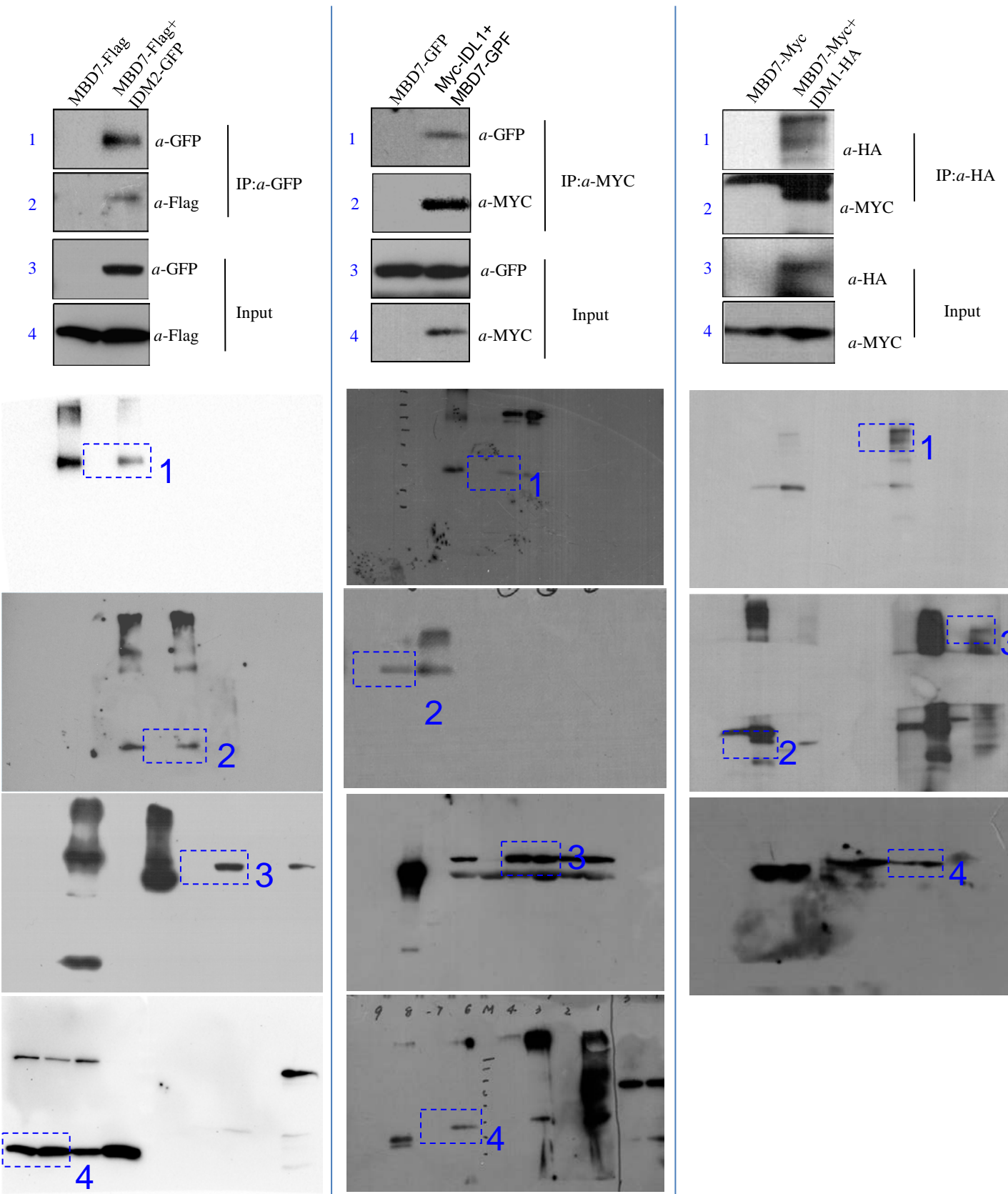

**Figure S8**

Supplement: S8 Fig — (PDF) [file pgen.1005210.s008.pdf]
