# Supplementary material for: Regulation of Active DNA Demethylation by a Methyl-CpG-Binding Domain Protein in Arabidopsis thaliana
Source: PLoS Genet. 2015 May 1;11(5):e1005210. doi: 10.1371/journal.pgen.1005210 (PMC4416881; doi:10.1371/journal.pgen.1005210)
Supplement: S7 Fig — (A) Mass-spectrometric analysis of IDM1 and IDL1 co-purifying proteins. (B) Split-luc assays showing that MBD6 can interact with IDM2 and IDL1 in N. benthamiana leaves. Three biological replicates were performed, and similar results were obtained. (C) Yeast two-hybrid assays. (D) Chop-PCR assay determine the DNA methylation level. Genomic DNA from wild type and mutant plants were digested with McrBC, a DNA methylation-dependent restriction enzyme. (PDF) [file pgen.1005210.s007.pdf]

A

IDM1 Purification

Protein Accession	Gene Accession	Protein	Score	Mass	Spectra	Unique Peptides	% Seq. Cov	% of IDM1
IPI00540986	AT3G14980	IDM1	4599	133697	236	52	39	100
IPI00545650	AT1G20870	IDL1	925	52470	36	16	39.7	81.5
IPI00531355	AT1G54840	IDM2	531	39712	42	12	37.2	80.8
IPI00524605	AT5G59800	MBD7	749	35127	40	12	40.8	83.7
IPI00536761	AT5G59380	MBD6	52	24435	1	1	5.2	-

IDL1 (IDM2 Like 1) Purification 2#

Protein Accession	Gene Accession	Protein	Score	Mass	Spectra	Unique Peptides	% Seq. Cov	% of IDL1
IPI00545650	AT1G20870	IDL1	535	52470	32	13	26.8	100
IPI00536761	AT5G59380	MBD6	212	24435	9	6	31.1	99.1

B

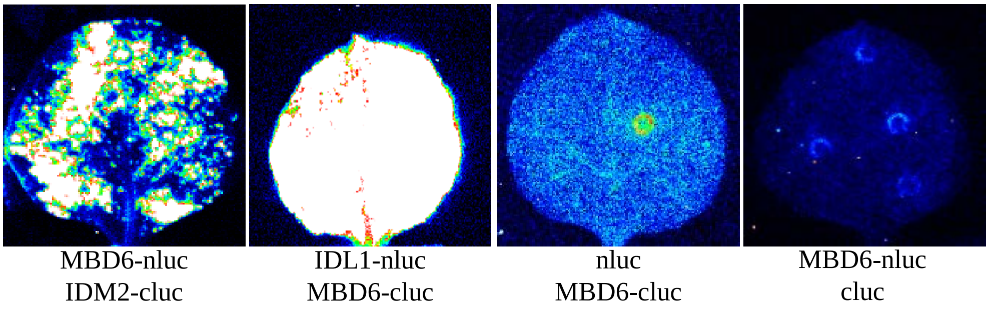

C

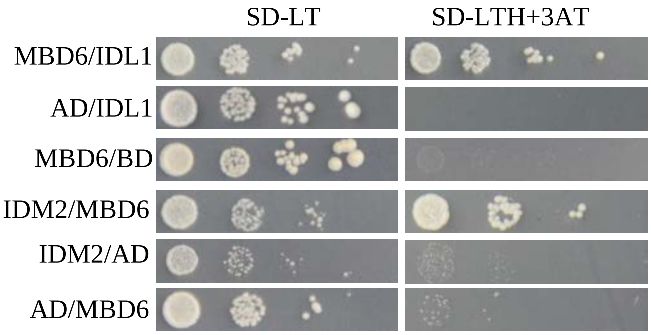

D

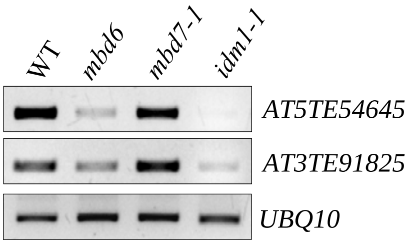

Figure S7
