# Supplementary material for: Regulation of Active DNA Demethylation by a Methyl-CpG-Binding Domain Protein in Arabidopsis thaliana
Source: PLoS Genet. 2015 May 1;11(5):e1005210. doi: 10.1371/journal.pgen.1005210 (PMC4416881; doi:10.1371/journal.pgen.1005210)
Supplement: S4 Table — (DOCX) [file pgen.1005210.s012.docx]

**Table S4. Primers used in this study.**

| Primer name | Primer sequence (5'-3') | Purpose |
| --- | --- | --- |
| *At1g66770*-F | AAAAGTTTGGAGTTCATGCCG | Real-time PCR |
| *At1g66770*-R | CAGGAATGGATCAAAAGGCAC |  |
| *At1g66780*-F | ACTTCTCTTACCAATGTCACCG | Real-time PCR |
| *At1g66780*-R | CATTGAGCCGTAAGTGTATGC |  |
| *At2g15410*-F | CTCCTGGTTTGAATTTGCCG | Real-time PCR |
| *At2g15410*-R | CTCGCAATGTATAGGTTCCCC |  |
| *At2g15420*-F | TTTCCTCGCTTCAGTCTTCC | Real-time PCR |
| *At2g15420*-R | GTGACTTCCTCAACCTAGCAC |  |
| *At4g20790*-F | CTCTCAGTATTCTCCGCAACC | Real-time PCR |
| *At4g20790*-R | AGCATTGGATCACGAAGACAG |  |
| *At4g20800*-F | GAAAGTACGGAATCTCGGTCG | Real-time PCR |
| *At4g20800*-R | ACGAGAGGATAACGCCAAAG |  |
| *At2g11005*-F | CAGTGTCTCCAGCGATAGTTC | Real-time PCR |
| *At2g11005*-R | ACATCACCAGAGCAACCAC |  |
| *At2g40420*-F | TCGTGCAAGGGAAGACAAAG | Real-time PCR |
| *At2g40420*-R | AGTTCGAATGCTACTGGATGG |  |
| *At1g34600*-F | AACATTAGGTGGGCTGAAGAG | Real-time PCR |
| *At1g34600*-R | TTCATCTCGTTCTCTGCCTTG |  |
| *At1g75950*-F | TCGAGTCACAAACCATAGCG | Real-time PCR |
| *At1g75950*-R | AATACTCGATCACCTTGGCG |  |
| *At3g62470*-F | GGCGAGAAACTATGAGATGGG | Real-time PCR |
| *At3g62470*-R | ACTTGCCTTCACCTATAAGCC |  |
| *At3g62475*-F | CGACTCCAAGATACCCTCAAG | Real-time PCR |
| *At3g62475*-R | CGAGGCGTAGTTCTGTATGAC |  |
| *At3g62480*-F | GACGCCTTCTCTATTCTACCTTG | Real-time PCR |
| *At3g62480*-R | GGCATTGATCTTGGTTGACG |  |
| *At3g62490*-F | GGAGAAAGGGAAAGGAGTGG | Real-time PCR |
| *At3g62490*-R | CGAAACGTCTTTGGAGCAAC |  |
| *LUC*-F | AAGCGAAGGTTGTGGATCTGGA | Real-time PCR |
| *LUC*-R | TCAATCAAGGCGTTGGTCGCTT |  |
| *NPTII*-F | ACCTTGCTCCTGCCGAGAAAGTAT | Real-time PCR |
| *NPTII*-R | ATGCGATCTTTCGCTTGGTGGT |  |
| DT-65-F | TGACTAGCTTTATGGACAAAATTGC | ChIP q-PCR |
| DT-65-R | TCGACTACCCTAAAACTTCAACG |  |
| DT-214-F | TATGGGAGGAGGAGGCCTAT | ChIP q-PCR |
| DT-214-R | TTTCAATGACTTTGCGCTTG |  |
| DT-265-F | TGAATTTTCCCTGGAGTTGC | ChIP q-PCR |
| DT-265-R | CAAAGCTTCAGCTGGGAAGT |  |
| DT-414-F | GGCATCGAAAAAGGGAATTT | ChIP q-PCR |
| DT-414-R | TGAACATTGTCGGAGCAAAA |  |
| DT-678-F | GCGGTGTTCATAGGGTCGTA | ChIP q-PCR |
| DT-678-R | CTCCGACAACATCTCCGACT |  |
| *At1g01260*-F | TCCGAATAATGGGAACAGGT | ChIP q-PCR |
| *At1g01260*-R | GGCCTCGTGTAGTTCTCAGG |  |
| *At1g10950*-F | GAGGAGCAGGTCACCCTATG | ChIP q-PCR |
| *At1g10950*-R | ACCACCCCATTTGTGAACAT |  |
| *Actin7*-F | CGGGCTAATTCATTTGAACC | ChIP q-PCR |
| *Actin7*-R | GGTGACCTGGCTTTCACACT |  |
| *At1g26400*-F1 | TGACCTGCATAGGCTATAACACA | Chop PCR |
| *At1g26400*-R1 | ATTGGAATCAATCCGAGTGG |  |
| *AT1G26410*-F | ATTCAACAGTAGCGTCTCGT | Chop PCR |
| *AT1G26410*-R | TCAATCTCTTCAAATTCCCC |  |
| *AT1G26390*-F | GAGTCCAGGAGATTTTTTCA | Chop PCR |
| *AT1G26390*-R | TTAGCTTCATCCACGTTTGT |  |
| *AT1G26380*-F | AAGGACTGGAGAAGATTTGG | Chop PCR |
| AT1G26380-R | CTCTGTTCGTTTTTGAAGAA |  |
| AT5G54020-F | CTTGTTAAGGTTAGTCACCT | Chop PCR |
| AT5G54020-R | CCACACTTCTCTTCTTGTTG |  |
| AT5G54030-F | ATTGCGTCTATGGAGGTTAT | Chop PCR |
| AT5G54030-R | GAGTGAGTTTATGGTTGTGC |  |
| AT5G54040-F | GAGCATAGTCTCACCCTCTT | Chop PCR |
| AT5G54050-R | TTAGCATCATCCGTTACATA |  |
| *MBD7*-F1 | AATTCTAGAATGCAGACGAGATCCTCTTC | Genotyping & RT-PCR |
| *MBD7*-R1 | AATGGTACCAGAGCGGTCTTCGATCAGTG |  |
| *MBD7*-F2 | TTTGAATTCCTGGAAATTCAGGTCCTAAGTCTG | Genomic DNA cloning |
| *MBD7*-R2 | TTTGGTACCAGAGCGGTCTTCGATCAGTGAAACAA |  |
| *MBD6-F1* | ATGTCAGATTCTGTGGCCGGC | Genotyping & RT-PCR |
| *MBD6-R1* | TCAAGCCGACACTTTACTAG |  |
| *AT5TE54645-F* | ATTCTTAGAGCGACAAAAACGC | Chop PCR |
| *AT5TE54645-R* | GTTTAACGCCAAAAGTCATAACA |  |
| *AT3TE91825-F* | GCGGATATTAGCACTAAACCCA | Chop PCR |
| *AT3TE91825-R* | TGAAAAGTATTTTGTTTAAAACGC |  |
